# On the Role of Entropy Flux and Entropy Production in the Modeling of Shape Memory Alloys

**DOI:** 10.3390/e28090998

**Published:** 2026-09-07

**Authors:** Claudio Giorgi, Angelo Morro

**Affiliations:** 1 Dipartimento di Ingegneria Civile Ambiente Territorio Architettura e Matematica, Università di Brescia, Via D. Valotti 9, 25133 Brescia, Italy; 2 Dipartimento di Informatica, Bioingegneria, Robotica e Ingegneria dei Sistemi, Università di Genova, Via All’Opera Pia 13, 16145 Genova, Italy; angelo.morro@unige.it

**Keywords:** second law of thermodynamics, Clausius–Duhem inequality, entropy flux, entropy production rate, shape memory alloys, 74A15, 74A20, 74N30, 74F05, 80A17

## Abstract

A thermodynamically consistent model of shape memory alloys is developed for a body in a uniaxial setting under a tensile stress. The evolution properties are described using the temperature, the martensite fraction, and the stress as independent variables. The innovative approach is based on a general form of the Clausius–Duhem inequality (really, an equality) where the entropy flux and the entropy production rate are given by constitutive functions. Thermodynamic restrictions and a suitable splitting of the entropy and deformation functions transform the Clausius–Duhem inequality into an evolutionary partial differential equation. As a result, both temperature-induced and stress-induced phase transitions and their related hysteretic loops are carefully modelled by properly choosing the free energy, dynamic functions, and the entropy production rate. Furthermore, a region of equilibrium states follows from a stationary condition on the free energy. Next, a generalization is given by letting the constitutive function depend on appropriate gradients within a Lagrangian and an Eulerian formulation. Both formulations are allowed by the occurrence of the extra-entropy flux that turns out to be proportional to the pertinent rates of temperature, stress, and mass fraction.

## 1. Introduction

Shape memory alloys (SMAs) are materials that exhibit a coupled thermomechanical behavior associated with hysteretic properties. This paper is devoted to the modeling of SMAs and is based on a thermodynamic approach where a general view is applied to entropy flux and entropy production.

The second law of thermodynamics represents the balance of entropy. In continuum thermodynamics, the local form of the balance was first established as

(1) $$\rho \dot{\eta }+\nabla \cdot \left(q/\theta \right)-\rho r=\rho \gamma ,$$

where η is the specific entropy density, ρ is the mass density, θ is the absolute temperature, q is the heat flux, *r* is the heat supply and γ the (specific rate of) entropy production. As a balance law, Equation (1) may be used to determine the entropy production in a given process. In addition to the balance of entropy, in Rational Thermodynamics (see, e.g., [1]) the second law has become the basic tool for the analysis of thermodynamic consistency of a material model. This role of the second law traces back to the Postulate laid down by Coleman and Noll [2], whereby *an admissible model of material makes γ≥0 for any process satisfying the balance equations.*

Next Müller [3] generalized the balance of entropy in the form

(2) $$\rho \dot{\eta }+\nabla \cdot j-\rho r=\rho \gamma ,$$

and then avoiding the assumption that the entropy flux j equals q/θ. Indeed, j is taken as a constitutive function to be determined on the basis of the Postulate. It is widely accepted that Equation (2), or Equation (1), holds with the entropy production γ being identically equal to the left-hand side. Both properties of (2) (and of (1)) hold as the balance of entropy and a thermodynamic censorship of admissible models by letting Equation (2) be satisfied as an equality and not an identity. Indeed, the role of Equation (2) as an equality, not necessarily an identity, proved essential in the modeling of hysteresis where eventually the left-hand side resulted as a combination of rates and not as a positive-definite expression. This has been the case of models of hysteretic (mechanical [4], ferromagnetic [5], ferroelectric [6]) materials and hence we let the entropy production γ be a constitutive equation that satisfies the balance of entropy and the Postulate. Furthermore, as is widely shown in the literature, the case j≠q/θ happens when non-local theories are developed through suitable gradients of the pertinent variables (see, e.g., [7,8,9,10] and refs therein).

With this general view on the thermodynamic consistency of continuum models, we regard SMAs as solid mixtures where the deformation and the composition are affected by both temperature and stress [11,12]. Hence, SMAs are described by temperature and stress and we look for models that provide the composition (or mass fraction) and the deformation. Furthermore, since the deformation is not isochoric, the consistency of the model requires a characterization of the mass density.

Also, with reference to standard experimental conditions, namely wire-like continua, we pay particular attention to a uniaxial setting. This allows us to characterize the stress in a scalar way and complete the three-dimensional geometry by letting the specific volume be related to temperature (thermal expansion). The joint dependence of constitutive functions on temperature, stress, and mass fraction leads to a form of the second law as a rate equation that describes the evolution of the specimen. The thermodynamic analysis of the rate equation, involving a thermodynamic potential, the entropy flux, and the entropy production is the main subject of this paper.

## 2. Notation and Balance Equations

The body occupies a time-dependent three-dimensional region Ω. Indeed, Ω is in a parallelepiped form Ω={I×S} where *I* is a segment and *S* is a square orthogonal to *I*. The position vector of a point in Ω is denoted by x. The motion is specified by a function x=χ(X,t), where X is the position vector of the point in a reference configuration R and t∈R is the time. Hence R∋X↦x∈Ω. For any function f(x,t), the symbol $\partial_{t}f$ is the partial time derivative, at constant x, while $\dot{f}$ is the time derivative at constant X. For any second-order tensor A, the symbols symA and skwA denote the symmetric and skew-symmetric parts of A. The symbols *∇* and $\nabla_{\;\;R}$ denote the gradient in Ω and R. We let F denote the deformation gradient, v the velocity, and L the velocity gradient, $F_{iK}=\partial_{X_{K}}\chi_{i}$, $v_{i}=\partial_{t}\chi_{i}\left(X,t\right)$, $L_{ij}=\partial_{x_{j}}v_{i}$. The Jacobian J=detF is assumed to be positive.

Let ρ(x,t) be the mass density in Ω and $\rho_{R}$ the mass density in R. The conservation of mass implies that

$$\rho J=\rho_{R}$$

where $\rho_{R}=\rho_{R}\left(X\right)$. In dealing with the behavior of alloys, we need a variable specifying the composition. It is standard to use a mass fraction, say ξ. For definiteness, we look at NiTi, with austenite and martensite constituents, and ξ is the martensite fraction. The mass density ρ is in fact given by

$$\rho =\rho_{A}\left(1-\xi \right)+\rho_{M}\xi ,$$

where $\rho_{A},\rho_{M}$ are the mass densities of austenite and martensite. Since $\rho_{A}=7.908$ g cm^−3^, $\rho_{M}=7.728$ g cm^−3^, with $\rho_{M}$ being slightly dependent on the type of martensite [13], then

$$\frac{1}{\rho_{A}}\frac{d\rho }{d\xi }=\frac{\rho_{M}-\rho_{A}}{\rho_{A}}≃2\cdot 10^{-3}.$$

We then follow the approximation that ρ is independent of ξ. Consistently, we identify $\rho_{R}$ with the uniform mass density of austenite in the reference configuration.

The composition of the alloy might be specified by further internal variables, or parameters, related to microscopic structural properties. Accounting for these properties would lead to the selection of particular functions characterizing the alloy (see G,S,Σ of Section 5). Hereafter we restrict attention to the pseudoelastic behaviour; when mechanically loaded, the alloy deforms reversibly to very high strains. For a given SMA composition, pseudoelasticity is observed in a finite temperature range (typically, around 50 °C). Within this window, the applied stress can induce martensite. According to Brinson’s model [14], the lower end is just above $A_{s}$, the temperature at which a stress-free alloy starts in austenite. The upper end is often denoted $M_{d}$; above $M_{d}$, the austenite is so stable that only plastic yielding (slip) occurs under load.

Let T denote the Cauchy stress tensor and b the body force per unit mass. The balance of linear momentum leads to the equation of motion in the form

$$\rho \dot{v}=\nabla \cdot T+\rho b,$$

where ${\left(\nabla \cdot T\right)}_{i}=\partial_{x_{j}}T_{ij}$. The balance of angular momentum implies that T is symmetric, $T=T^{T}$.

Let D=symL be the stretching tensor. Denote by ε the internal energy density per unit mass, q the heat flux vector and *r* the energy supply. The balance of energy results in the local equation

(3) $$\rho \dot{\varepsilon }=T\cdot D-\nabla \cdot q+\rho r.$$

Let θ be the absolute temperature, η the entropy density per unit mass, j the entropy flux, and r/θ the assumed entropy supply. The balance of entropy leads to the local equation

$$\rho \dot{\eta }+\nabla \cdot j-\frac{\rho r}{\theta }=\rho \gamma ,$$

where γ is the (rate of) entropy production.

Motivated by the experimental setting for SMAs subject to a tensile stress, we simplify the description by letting *I* be the segment of length l(t), in the direction $e_{1}$. Let *x* denote the coordinate along the axis of *I* and X∈[0,l(0)] the coordinate in the reference interval. Let $\sigma =T_{11}$ be the applied traction in the $e_{1}$ direction and hence we assume

$$T\left(x,t\right)=\sigma \left(x,t\right)e_{1}⊗e_{1}.$$

On the lateral surface of the parallelepiped, it is Tn=0. We assume the deformation gradient F is diagonal and we let

$$F=\mathrm{diag}[F,F_{⊥},F_{⊥}],$$

where $F=\partial_{X}x$ and $F_{⊥}$ is the common value of F at the lateral surface. Since σ>0 then we assume F>0 whence

$$J=FF_{⊥}^{2}>0.$$

The values of *F* and $F_{⊥}$ depend on σ and also on θ. While *F* is an essential unknown of the problem, we point out that the Jacobian *J* is subject to $\dot{J}=J\nabla \cdot v$. In detailed models, *J* and ρ are viewed as constants (see, e.g., [15] chapter 3). Here we allow *J* to be affected by thermal expansion and let

J=J(θ)

whence $\rho =\rho \left(\theta \right)=\rho_{R}/J\left(\theta \right)$. The dependence on thermal expansion is consistent with the boundary condition Tn=0 at the lateral surface. Next we point out how to proceed in the more general case where J=J(θ,σ).

Owing to the uniaxial setting, we observe that the power T·D takes a simple form. Since $L=\dot{F}F^{-1}$ then

$$T\cdot D=\sigma L_{11}=\sigma F^{-1}\dot{F}=\sigma \dot{\delta },\;\delta :=\ln F.$$

Hence the balance of energy (3) can be written in the form

(4) $$\rho \dot{\varepsilon }=\sigma \dot{\delta }-\nabla \cdot q+\rho r.$$

Since $FF_{⊥}^{2}=J$ then the whole deformation gradient is determined by δ and θ in that

$$F_{⊥}={\left(J\left(\theta \right)/F\right)}^{1/2},\;F=\exp\delta .$$

## 3. Thermodynamic Scheme for SMAs

A process P is the set of fields entering the balance equations; here P is the set

(χ,θ,ρ,T,q,ε,η,j,γ,b,r).

As conducted in recent applications of ours [16], we let γ be given by a constitutive function. Following [2] and subsequent generalizations [3,6], we consider the second law as a material selection in that physically admissible sets of constitutive functions are required to satisfy the following

**Postulate** **1.** For every process P admissible in a body the inequality

(5) $$\rho \dot{\eta }+\nabla \cdot j-\frac{\rho r}{\theta }=\rho \gamma \geq 0$$

is valid at any internal point.

Henceforth, Equation (5) is referred to as the CD (Clausius–Duhem) inequality. Let j=q/θ+k and regard k as the extra-entropy flux. Substituting ∇·q−ρr from (4) into (5) we obtain

(6) $$-\rho \left(\dot{\psi }+\eta \dot{\theta }\right)+\sigma \dot{\delta }+\theta \nabla \cdot k-\frac{1}{\theta }q\cdot \nabla \theta =\rho \theta \gamma ,$$

where ψ=ε−θη is the Helmholtz free energy, per unit mass. To save writing, henceforth it is understood and not written that γ≥0.

Since we are interested in the response of the material to the applied stress σ, it is convenient to change the form (6) of the CD inequality to the terms in $\dot{\delta }$ and $\dot{\theta }$. First we multiply Equation (6) by *J* and notice that $\rho J=\rho_{R}$ to obtain

$$-\rho_{R}\left(\dot{\psi }+\eta \dot{\theta }\right)+J\sigma \dot{\delta }+J\theta \nabla \cdot k-\frac{J}{\theta }q\cdot \nabla \theta =\rho_{R}\theta \gamma .$$

Using the identity

$$J\sigma \dot{\delta }=\dot{\left(J\sigma \delta \right)}-\delta \sigma \dot{J}-\delta J\dot{\sigma }$$

and observing that

$$\dot{J}=J^{\prime }\dot{\theta }$$

we can write the CD inequality in the form

(7) $$-\rho_{R}\left(\dot{\phi }+\tilde{\eta }\dot{\theta }\right)-\delta J\dot{\sigma }+J\theta \nabla \cdot k-\frac{J}{\theta }q\cdot \nabla \theta =\rho_{R}\theta \gamma ,$$

where

$$\phi =\psi -\frac{1}{\rho_{R}}J\sigma \delta ,\;\tilde{\eta }=\eta +\frac{1}{\rho_{R}}\delta \sigma J^{\prime }.$$

The function ϕ represents the Gibbs free energy, per unit mass.

In the modeling of hysteretic materials, the stress or the deformation is decomposed additively, e.g.,

(8) $$\delta =\delta^{el}+\delta^{dyn},$$

with “el” viewed as energetic or elastic and “dyn” as dissipative or transition parts [17,18,19]. Similar decompositions are established in this paper as a consequence of the CD inequality (7).

### Thermodynamic Restrictions

Let ξ be the mass fraction of an SMA constituent; for NiTi, we let ξ be the martensite fraction. The evolution of ξ is assumed to be unaffected by the spatial derivatives ∇ξ,∇∇ξ. The temperature gradient ∇θ is also considered to allow for heat conduction. Accordingly, we describe the alloy by letting

$$\phi ,\eta ,\delta ,\dot{\xi },k,q,\gamma$$

be constitutive functions of the variables

$$\theta ,\sigma ,\xi ,\dot{\theta },\nabla \theta ,\dot{\sigma }.$$

For simplicity, the free energy ϕ is taken to be independent of ∇θ. A possible dependence of ϕ on ∇θ and ∇ξ is investigated in Section 6.

Computation of $\dot{\phi }$ and substitution in (7) result in

$$\begin{array}{c} -\rho_{R}\left(\partial_{\theta }\phi +\tilde{\eta }\right)\dot{\theta }-\left(\rho_{R}\partial_{\sigma }\phi +J\delta \right)\dot{\sigma }-\rho_{R}\partial_{\xi }\phi \;\dot{\xi }-\rho_{R}\partial_{\dot{\theta }}\phi \;\ddot{\theta }\\ -\rho_{R}\partial_{\dot{\sigma }}\phi \;\ddot{\sigma }+J\theta \nabla \cdot k-\frac{J}{\theta }q\cdot \nabla \theta =\rho_{R}\theta \gamma . \end{array}$$

The linearity and arbitrariness of $\ddot{\theta }$ and $\ddot{\sigma }$ imply that

$$\partial_{\dot{\theta }}\phi =0,\;\partial_{\dot{\sigma }}\phi =0.$$

Since $k=k(\theta ,\sigma ,\xi ,\dot{\theta },\nabla \theta ,\dot{\sigma })$ then

$$\nabla \cdot k=\partial_{\theta }k\cdot \nabla \theta +\partial_{\sigma }k\cdot \nabla \sigma +\partial_{\xi }k\cdot \nabla \xi +\partial_{\dot{\theta }}k\cdot \nabla \dot{\theta }+\partial_{\nabla \theta }k\cdot \nabla \nabla \theta +\partial_{\dot{\sigma }}k\cdot \nabla \dot{\sigma }$$

The linearity and arbitrariness of $\nabla \sigma ,\nabla \xi ,\nabla \dot{\theta },\nabla \nabla \theta ,\nabla \dot{\sigma }$ imply that $\partial_{\theta }k$, $\partial_{\sigma }k,$$\partial_{\xi }k,\partial_{\dot{\theta }}k,\partial_{\nabla \theta }k$, and $\partial_{\dot{\sigma }}k$ are required to vanish. Accordingly, k might depend only on θ. However, in isotropic bodies no vector k can be determined by the scalar θ. Hence, we let k=0. Consequently, the CD inequality simplifies to

(9) $$-\rho_{R}\left(\partial_{\theta }\phi +\tilde{\eta }\right)\dot{\theta }-\left(\rho_{R}\partial_{\sigma }\phi +J\delta \right)\dot{\sigma }-\rho_{R}\partial_{\xi }\phi \;\dot{\xi }-\frac{J}{\theta }q\cdot \nabla \theta =\rho_{R}\theta \gamma .$$

with

(10) ϕ=ϕ(θ,σ,ξ).

Further restrictions are derived as admissible conditions or as consequences of simple assumptions. Equation (9) can be viewed as a relation between $\dot{\theta },\dot{\sigma }$, and $\dot{\xi }$ affected by the entropy production γ and the heat flux q or as the constitutive equation for the rate $\dot{\xi }$. As a possible restriction, we assume $\dot{\xi }$ is independent of ∇θ. For definiteness we assume

$$\gamma =\gamma_{\xi }\left(\theta ,\sigma ,\xi ,\dot{\theta },\dot{\sigma }\right)+\gamma_{q}\left(\theta ,\sigma ,\xi ,\nabla \theta \right),\;\partial_{\nabla \theta }\dot{\xi }=0,$$

where $\gamma_{\xi }=0$ if $\dot{\theta }=0,\dot{\sigma }=0$ and $\gamma_{q}=0$ if ∇θ=0. Hence Equation (9) splits into

(11) $$-\rho \left(\partial_{\theta }\phi +\tilde{\eta }\right)\dot{\theta }-\left(\rho \partial_{\sigma }\phi +\delta \right)\dot{\sigma }-\rho \partial_{\xi }\phi \;\dot{\xi }=\rho \theta \gamma_{\xi },$$

(12) $$-\frac{1}{\theta }q\cdot \nabla \theta =\rho \theta \gamma_{q}.$$

In light of (11) we observe that, for the sake of generality, $\tilde{\eta }$ and δ are allowed to be partly dissipative in that

(13) $${\tilde{\eta }}^{dyn}:=\tilde{\eta }+\partial_{\theta }\phi ,\;\delta^{dyn}:=\delta +\rho \partial_{\sigma }\phi$$

are not identically zero and then Equation (11) takes the form

(14) $$-\rho {\tilde{\eta }}^{dyn}\dot{\theta }-\delta^{dyn}\dot{\sigma }-\rho \partial_{\xi }\phi \;\dot{\xi }=\rho \theta \gamma ;$$

hereafter γ stands for $\gamma_{\xi }$. Hence $\tilde{\eta }$ and δ are expressed by an additive decomposition,

(15) $$\tilde{\eta }=-\partial_{\theta }\phi +{\tilde{\eta }}^{dyn},\;\delta =-\rho \partial_{\sigma }\phi +\delta^{dyn}.$$

Since ϕ is a function of θ,σ,ξ then Equation (11) can be written in the form

(16) $$-\rho \left(\dot{\phi }+\tilde{\eta }\dot{\theta }\right)-\delta \dot{\sigma }=\rho \theta \gamma .$$

Equations (14)–(16) are the basic relations for the modeling of SMAs.

By way of comment, we observe that, upon the definitions

$${\tilde{\eta }}^{el}:=-\partial_{\theta }\phi ,\;\delta^{el}:=-\rho \partial_{\sigma }\phi ,$$

we can express $\tilde{\eta }$ and δ through the additive decompositions (8) in the form

$$\tilde{\eta }={\tilde{\eta }}^{el}+{\tilde{\eta }}^{dyn},\;\delta =\delta^{el}+\delta^{dyn},$$

where ${\tilde{\eta }}^{dyn}$ and $\delta^{dyn}$ are independent constitutive functions to be determined subject to (14). Furthermore, by (15) we can write the physical entropy η in the form

$$\eta =\eta^{el}+\eta^{dyn},\;\eta^{el}:=-\partial_{\theta }\phi +\frac{J^{\prime }}{J}\sigma \partial_{\sigma }\phi ,\;\eta^{dyn}={\tilde{\eta }}^{dyn}-\frac{1}{\rho_{R}}\delta^{dyn}\sigma J^{\prime }.$$

Since ϕ is a function of θ,σ,ξ so are $\delta^{el}$ and $\eta^{el}$. Hence the physical entropy η is related to ϕ through $\eta^{el}$ while $\eta^{dyn}$ arises from the dynamic parts ${\tilde{\eta }}^{dyn}$ and $\delta^{dyn}$.

While $\eta^{el}$ and $\delta^{el}$ are determined by the free energy ϕ and the function J(θ), the dynamic parts $\eta^{dyn}$ and $\delta^{dyn}$ are affected by the dissipative terms in the CD inequality. Accordingly, it is of interest to determine the internal energy ε. We find that

$$\varepsilon =\phi +\theta \left(\eta^{el}+\eta^{dyn}\right)-\frac{1}{\rho }\sigma \left(\delta^{el}+\delta^{dyn}\right)=\phi +\theta \left(-\partial_{\theta }\phi +\eta^{dyn}\right)-\frac{J(\theta )}{\rho_{R}}\sigma \left(-\rho \partial_{\sigma }\phi +\delta^{dyn}\right),$$

where

$$\eta^{dyn}={\tilde{\eta }}^{dyn}-\frac{1}{\rho_{R}}\delta^{dyn}\sigma J^{\prime }$$

while ${\tilde{\eta }}^{dyn}$ and $\delta^{dyn}$ are the dynamic terms chosen to satisfy the CD inequality (14).

**Remark** **1.** *For a greater generality we might allow ρ to depend also on the stress σ. In that case J=J(θ,σ) and then $\delta J\dot{\sigma }$ in (7) would be modified to*

$$\delta J\dot{\sigma }\mapsto \delta \left(J+\sigma \partial_{\sigma }J\right)\dot{\sigma }$$

*while $\tilde{\eta }=\eta +\delta \sigma \partial_{\theta }J/\rho_{R}$. This would lead to*

$$\delta^{dyn}=\rho \partial_{\sigma }\phi +\delta \left(1+\sigma \partial_{\sigma }J/J\right)$$

*whence*

$$\delta =\frac{1}{1+\sigma \partial_{\sigma }J/J}\left[-\rho \partial_{\sigma }\phi +\delta^{dyn}\right]$$

*Consequently, the dependence of J on σ would produce higher-order terms in σ (see Section 5.4) which do not seem to be motivated on the experimental side.*

## 4. Properties of Cycles in the (θ,σ,ξ) Space

Equation (16) may be viewed as an anholonomic constraint on the evolution of the time-dependent functions $\theta \left(t\right),\sigma \left(t\right),\xi \left(t\right),\phi \left(t\right),\tilde{\eta }\left(t\right),\delta \left(t\right),\gamma \left(t\right)$. Cycles, as t∈[0,T], are particular solutions to (16) that satisfy the closure condition ξ(T)=ξ(0) and the like for the other functions. For definiteness, we restrict attention to cycles at constant temperature or at constant stress.

Let θ be constant (isothermal process) and consider the curve (σ(t),ξ(t)) in the σ−ξ plane with the closure conditions σ(T)=σ(0),ξ(T)=ξ(0). Since $\phi \left(t\right)=\hat{\phi }\left(\sigma \left(t\right),\xi \left(t\right)\right)$ then also ϕ satisfies the closure condition, ϕ(T)=ϕ(0). Meanwhile, no assumption is made about δ and γ except the thermodynamic requirement γ≥0.

Since $\dot{\theta }=0$ then Equation (16) simplifies to

(17) $$-\rho \dot{\phi }-\delta \dot{\sigma }=\rho \theta \gamma ,$$

where ρ is constant. Integration of (17) on [0,T] yields

(18) $$\int_{0}^{T}\delta \dot{\sigma }\;dt=-\rho \theta \int_{0}^{T}\gamma \left(t\right)dt\leq 0.$$

Consider a closed curve $C_{\sigma \delta }$ in the stress-strain plane, $C_{\sigma \delta }:t\mapsto \left(\sigma \left(t\right),\delta \left(t\right)\right)$, t∈[0,T], with the closure conditions σ(T)=σ(0),δ(T)=δ(0). Thus we have

(19) $$\int_{C_{\sigma \delta }}\delta \;d\sigma =\int_{0}^{T}\delta \;\dot{\sigma }\;dt\leq 0.$$

To within the sign, the line integral on $C_{\sigma \delta }$ is the area bounded by $C_{\sigma \delta }$. Since the integral turns out to be negative, it follows that the curve $C_{\sigma \delta }$ is oriented in the counterclockwise sense and

$$A=\rho \theta \int_{0}^{T}\gamma \left(t\right)dt$$

is the positive are bounded by $C_{\sigma \delta }$ in the σ−δ plane. In the more natural strain-stress δ−σ plane, the overturned closed curve $C_{\delta \sigma }$ is oriented in the clockwise sense.

As a comment on the condition δ(T)=δ(0) we notice that the closure conditions σ(T)=σ(0),ξ(T)=ξ(0) imply that of δ if

(20) $$\delta =\hat{\delta }\left(\theta ,\sigma ,\xi \right).$$

Owing to (15), the requirement (20) is a consistency restriction on $\delta^{dyn}$.

Accordingly, we have proved that, as a consequence of the second law, and subject to (20), the following statement is valid.

**Statement** **1.** *Cycles in the strain–stress δ−σ plane are oriented in the clockwise sense.*

**Figure 1:**
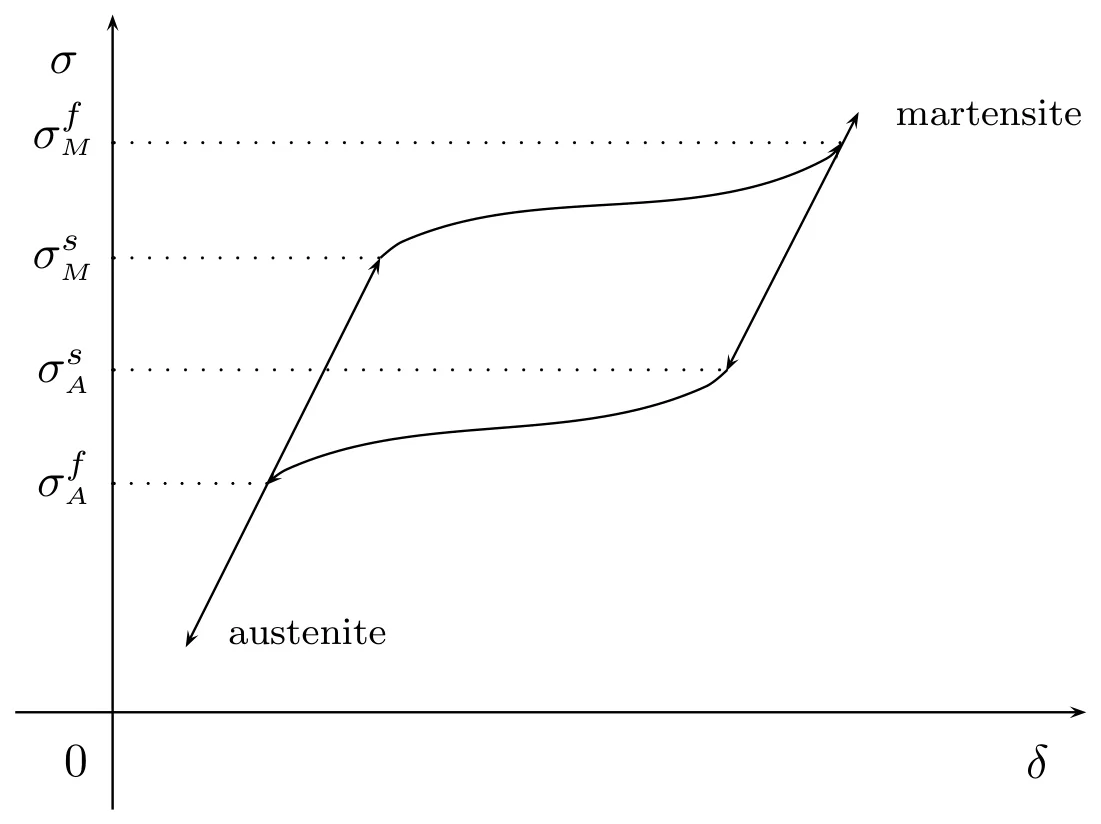
Schematic description of the stress-strain major hysteresis loop at constant temperature.

This statement is confirmed by experimental data. Figure 1 is considered for later convenience and is taken from Figures 1b, 9 and 10 of [17].

Cycles are dissipative processes; by (18) it follows that

$$\rho \theta \int_{0}^{T}\gamma \left(t\right)dt$$

is the power expended during any cycle. Reversible changes occur if ξ=0 or 1, as is shown next by Equation (24), though with a non-zero dissipation.

The orientation of cycles in the θ−ξ and σ−ξ planes is not a direct consequence of the second law. We then state two properties of cycles that are shown by experimental data.

**Statement** **2.** *Cycles in the θ−ξ plane are oriented in the clockwise sense.*

**Statement** **3.** *Cycles in the σ−ξ plane are oriented in the counterclockwise sense.*

Statement 2 is consistent with the experimental data, e.g., in [20] and [15] chapter 1, while Statement 3 is verified by hysteresis loops as, e.g., in Figure 3 of [21].

The clockwise orientation of cycles at constant temperature, in the δ−σ plane, follows from the second law whenever ϕ and δ are functions of θ,σ,ξ. Otherwise, we need the knowledge of the constitutive equations.

## 5. An Operative Scheme of Constitutive Equations for SMAs

The material properties of SMAs are described by the rate (anholonomic) Equations (14) and (16). Hence, definite properties may be derived once we establish the free energy ϕ, the dynamic functions ${\tilde{\eta }}^{dyn},\delta^{dyn}$, and the entropy production γ.

### 5.1. Gibbs Free Energy

Since SMAs are alloys of different metals, the starting point is often to regard the alloy as a solid mixture and then to consider the free energy (density) of the two constituents. Here too we follow this view and let

$$\phi_{A}=G\left(\theta ,\sigma \right),\;\phi_{M}=G\left(\theta ,\sigma \right)+D\left(\theta ,\sigma \right)$$

denote the (Gibbs) free energy of austenite and martensite. By the theory of mixtures, the free energy of a mixture equals the inner part plus a kinetic term representing the interaction between the constituents (see, e.g., [16], §9.4). Accordingly, we let the inner part $\phi_{I}$ be in the form

$$\phi_{I}\left(\theta ,\sigma ,\xi \right)=\left(1-\xi \right)\phi_{A}\left(\theta ,\sigma \right)+\xi \phi_{M}\left(\theta ,\sigma \right)=G\left(\theta ,\sigma \right)+\xi D\left(\theta ,\sigma \right).$$

Following [22], we let the interaction energy be given by a quadratic function of ξ, namely proportional to ξ(1−ξ). Accordingly, the free energy of the alloy is taken in the form

$$\phi \left(\theta ,\sigma ,\xi \right)=G\left(\theta ,\sigma \right)+\xi D\left(\theta ,\sigma \right)+\frac{1}{2}\alpha \xi \left(1-\xi \right).$$

It is worth mentioning that within phase-field theories (see, e.g., [23,24]) the inner part $\phi_{I}$ is viewed as the free energy of the mixture $\phi^{mix}$ while the interaction term $\frac{1}{2}\alpha \xi \left(1-\xi \right)$ is also denoted as $\phi^{exc}$ (exchange term) and is referred to as coherency. In general, coherency denotes the elastic energy stored within the material when two crystal phases maintain a continuous crystal lattice. Micro-mechanics indicates that this energy depends on temperature and possibly internal parameters. The present model allows for a further coherency effect via the term ξS(θ).

The difference D between $\phi_{M}$ and $\phi_{A}$ is taken to be expressed by an additive decomposition of two functions, S(θ) and Σ(σ). For technical convenience, we let

D(θ,σ)=S(θ)−Σ(σ)+p,

where *p* is a reference parameter. Hence it follows

$$\phi =G\left(\theta ,\sigma \right)+\xi \left[S\left(\theta \right)-\Sigma \left(\sigma \right)+p\right]+\frac{1}{2}\alpha \xi \left(1-\xi \right).$$

Since

$$\partial_{\xi }\phi =-\alpha \xi +S\left(\theta \right)-\Sigma \left(\sigma \right)+p+\frac{1}{2}\alpha ,$$

then using the insofar undefined S and Σ we choose p=−α/2. Accordingly we have

(21) $$\phi =G\left(\theta ,\sigma \right)+\xi \left[S\left(\theta \right)-\Sigma \left(\sigma \right)-\frac{1}{2}\alpha \right]+\frac{1}{2}\alpha \xi \left(1-\xi \right).$$

Hereafter, the free energy ϕ of the alloy is assumed in the form (21).

### 5.2. Dynamic Functions

The assumption (21) of ϕ and the definitions of ${\tilde{\eta }}^{el}$ and $\delta^{el}$ yield

(22) $$\begin{array}{cc} \delta^{el} & =-\rho \partial_{\sigma }\phi =-\rho \left[\partial_{\sigma }G\left(\theta ,\sigma \right)-\Sigma^{\prime }\left(\sigma \right)\xi \right],\\ {\tilde{\eta }}^{el} & =-\partial_{\theta }\phi =-\left[\partial_{\sigma }G\left(\theta ,\sigma \right)-\Sigma^{\prime }\left(\sigma \right)\xi \right], \end{array}$$

where $\rho =\rho_{R}/J\left(\theta \right)$.

To determine $\delta^{dyn}$ and ${\tilde{\eta }}^{dyn}$ we follow two requirements. First, as any constitutive function, $\delta^{dyn}$ and ${\tilde{\eta }}^{dyn}$ have to satisfy the CD inequality. The definition (13), constrained by the CD relation (14), makes $\delta^{dyn}$ and ${\tilde{\eta }}^{dyn}$ consistent with the thermodynamic restriction. Secondly, the term $\partial_{\xi }\phi \dot{\xi }$ in the CD inequality (or evolution equation) (16) indicates that $\partial_{\xi }\phi =0$ represents an equilibrium condition, associated with $\dot{\xi }=0$. Accordingly, since ${\tilde{\eta }}^{dyn}$ and $\delta^{dyn}$ model the effects of the temperature and/or stress evolution, we find it natural to assume that the dynamic functions ${\tilde{\eta }}^{dyn},\delta^{dyn}$ are proportional to $\partial_{\xi }\phi$. We then assume that

(23) $$\begin{array}{c} \delta^{dyn}=-\rho \varkappa \left(\sigma ,\xi \right)\partial_{\xi }\phi =\rho \varkappa \left(\sigma ,\xi \right)\left[\alpha \xi -S\left(\theta \right)+\Sigma \left(\sigma \right)\right],\;\varkappa >0,\\ {\tilde{\eta }}^{dyn}=\kappa \left(\theta ,\xi \right)\partial_{\xi }\phi =-\kappa \left(\theta ,\xi \right)\left[\alpha \xi -S\left(\theta \right)+\Sigma \left(\sigma \right)\right],\;\kappa >0. \end{array}$$

The positive definiteness of ϰ,κ implies that $\delta^{dyn}$ and ${\tilde{\eta }}^{dyn}$ are zero if and only if $\partial_{\xi }\phi$ is zero. Hence $\delta^{dyn}$ and ${\tilde{\eta }}^{dyn}$ are defined by (23), and subject to the CD inequality (14) whence

$$-\rho \kappa \partial_{\xi }\phi \;\dot{\theta }-\rho \varkappa \partial_{\sigma }\phi \;\dot{\sigma }-\rho \partial_{\xi }\phi \;\dot{\xi }=\rho \theta \gamma .$$

In Proposition 1 we will show how to satisfy the constraint ξ∈[0,1] by assuming some sufficient conditions on κ and ϰ.

Since $\delta =\delta^{el}+\delta^{dyn}$ and $\tilde{\eta }={\tilde{\eta }}^{el}+{\tilde{\eta }}^{dyn}$ then it follows that

(24) $$\delta =\rho \{-\partial_{\sigma }G\left(\theta ,\sigma \right)+\Sigma^{\prime }\left(\sigma \right)\xi +\varkappa \left(\sigma ,\xi \right)\left[\alpha \xi -S\left(\theta \right)+\Sigma \left(\sigma \right)\right]\},\;\tilde{\eta }=-\partial_{\theta }G\left(\theta ,\sigma \right)-S^{\prime }\left(\theta \right)\xi -\kappa \left(\theta ,\xi \right)\left[\alpha \xi -S\left(\theta \right)+\Sigma \left(\sigma \right)\right].$$

### 5.3. Equilibrium Surface

The dynamic functions (23) vanish when

$$\partial_{\xi }\phi =-\left[\alpha \xi -S\left(\theta \right)+\Sigma \left(\sigma \right)\right]=0.$$

We can then say that

(25) αξ−S(θ)+Σ(σ)=0, ξ∈[0,1],

characterizes the states of equilibrium of the system. Hence Equation (25) represents an *equilibrium surface*, say S, in the space of states (θ,σ,ξ).

For practical convenience consider an equilibrium state with ξ=0 (of austenite), say $\left(\hat{\theta },\hat{\sigma },0\right)$. By definition, the values $\hat{\theta },\hat{\sigma }$ are required to satisfy the condition

$$\Sigma \left(\hat{\sigma }\right)=S\left(\hat{\theta }\right).$$

Instead, in an equilibrium state with ξ=1, say $\left(\bar{\theta },\bar{\sigma },1\right)$, the values $\bar{\theta }$ and $\bar{\sigma }$ are subject to

$$\Sigma \left(\bar{\sigma }\right)=S\left(\bar{\theta }\right)-\alpha .$$

For definiteness, we let Σ be a monotone increasing, and then invertible, function. Hence we can consider the function $\Sigma^{-1}$ and define

$$T_{0}\left(\theta \right)=\Sigma^{-1}S\left(\theta \right),\;\tau_{\alpha }\left(\theta \right)=\Sigma^{-1}\left[S\left(\theta \right)-\alpha \right].$$

Thus we obtain two curves, $R_{0},R_{1}$, such that

$$R_{0}=\left\{\left(\theta ,\sigma ,\xi \right):\xi =0,\sigma =T_{0}\left(\theta \right)\right\},\;R_{1}=\left\{\left(\theta ,\sigma ,\xi \right):\xi =1,\sigma =T_{\alpha }\left(\theta \right)\right\}.$$

In particular, $\sigma =T_{0}\left(\theta \right)$ is the Clausius-Clapeyron relation that defines the critical stress required to induce a phase transformation between austenite and martensite. As temperature increases, higher mechanical stress is needed to trigger the crystal transformation; hence $T_{0}^{\prime }>0$. Usually, the critical stress is assumed to depend linearly on temperature (see [17]).

The function $q\left(\theta \right)=T_{\alpha }\left(\theta \right)-T_{0}\left(\theta \right)$ denotes the width of the equilibrium range at temperature θ. This value is parameterized by α and vanishes if α=0 and hence if the coherency is zero.

**Figure 2:**
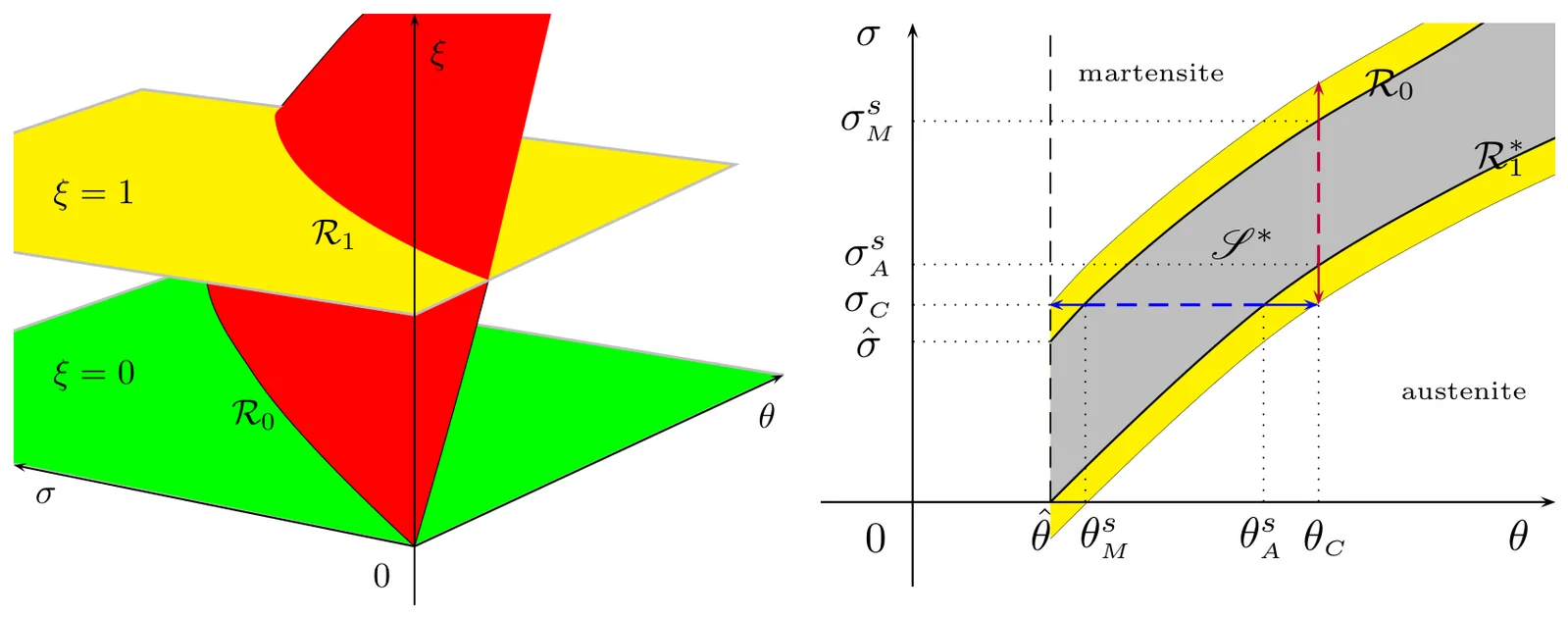
On the (**left**), schematic description of the equilibrium surface S (in red) and the planes ξ=0 (in green) and ξ=1 (in yellow). On the (**right**), schematic description of transition lines in the θ−σ plane; at constant stress $\sigma_{C}$ (blue) and constant temperature $\theta_{C}$ (purple).

Figure 2 (left) shows the equilibrium surface S in the (θ,σ,ξ)-space and its intersections $R_{0},R_{1}$ with the plane ξ=0,ξ=1, respectively.

Transition lines at constant stress and constant temperature are depicted in Figure 2 (right). The region $S^{*}$ (gray) represents the projection of the equilibrium surface S on the plane ξ=0, while $R_{1}^{*}$ is the projection of $R_{1}$ on the same plane. The yellow regions are the transformation strips. Moreover, $\hat{\theta }>0$ represents the starting temperature of an austenitic transformation during heating ($\theta_{A}^{s}$) in a stress-free state, usually denoted by $A_{s}$. The so-called *critical stress*, $\sigma^{\mathrm{cr}}$, is the stress at which martensitic transition starts. It is usually denoted by $\sigma_{M}^{s}$ and depends on temperature θ. Here $\hat{\sigma }$ represents the critical stress at $\theta =\hat{\theta }$.

### 5.4. Entropy Production

In the modeling of hysteretic materials (see, e.g., [4,12] and [16] chapter 15), the entropy production γ occurs in the CD inequality through its proper constitutive function and, accordingly, the CD inequality involves γ in the form of an equation, not an identity. To complete the thermodynamic scheme of SMAs, we then need to select the non-negative constitutive function of γ. In accordance with the assumptions (23) on $\delta^{dyn}$ and ${\tilde{\eta }}^{dyn}$, we notice that $\partial_{\xi }\phi$ is a common factor in the left-hand side of the reduced CD inequality (9),

(26) $$-\kappa \left(\theta ,\xi \right)\partial_{\xi }\phi \;\dot{\theta }+\varkappa \left(\sigma ,\xi \right)\partial_{\xi }\phi \;\dot{\sigma }-\partial_{\xi }\phi \;\dot{\xi }=\theta \gamma .$$

Equation (26) describes the evolution of θ,σ,ξ. If $\dot{\theta }=0,\dot{\sigma }=0$ then Equation (26) simplifies to

(27) $$-\partial_{\xi }\phi \;\dot{\xi }=\theta \hat{\gamma },$$

where $\hat{\gamma }$ is the restriction of γ at $\dot{\theta }=0,\dot{\sigma }=0$. Upon multiplication of Equation (27) by $\partial_{\xi }\phi$ we find

$$\dot{\xi }=-\frac{\theta \hat{\gamma }}{|\partial_{\xi }{\phi |}^{2}}\partial_{\xi }\phi .$$

Accordingly we let

$$\hat{\gamma }=\lambda \left(\theta \right){\left|\partial_{\xi }\phi \right|}^{2},\;\lambda >0.$$

To complete the expression of γ, we observe that $\partial_{\xi }\phi$ is a common factor in the left-hand side of (26) and hysteresis is induced by temperature or stress rates. Hence (θ times) the entropy production is assumed to have the form

(28) $$\theta \gamma =g\left(\xi \right)|\partial_{\xi }\phi |[\beta \left(\theta ,\xi \right)|\dot{\theta }\left|+\nu \left(\theta ,\xi \right)\right|\dot{\sigma }\left|]+\lambda \left(\theta \right)\right|\partial_{\xi }{\phi |}^{2},$$

where g(ξ)>0 if ξ∈[0,1] and β(θ,ξ),ν(σ,ξ),λ(θ) are positive-valued functions. Consequently, if $\partial_{\xi }\phi =0$ then all terms in the CD inequality (26) are zero. This is consistent with the view that $\partial_{\xi }\phi =0$ is the equilibrium condition.

Outside equilibrium, it is $\partial_{\xi }\phi \neq 0$ and the evolution of the system is given by the CD inequality (26). Upon dividing Equation (26) by $\partial_{\xi }\phi$ and using (28) we have

$$-\kappa \left(\theta ,\xi \right)\dot{\theta }+\varkappa \left(\sigma ,\xi \right)\dot{\sigma }-\dot{\xi }=\lambda \left(\theta \right)\partial_{\xi }\phi +g\left(\xi \right)\;\mathrm{sgn}\left(\partial_{\xi }\phi \right)\;[\beta \left(\theta ,\xi \right)|\dot{\theta }|+\nu \left(\sigma ,\xi \right)|\dot{\sigma }\left|\right].$$

More explicitly, since $\partial_{\xi }\phi =-\alpha \xi +S\left(\theta \right)-\Sigma \left(\sigma \right)$ then we can write

(29) $$\begin{array}{cc} -\kappa \left(\theta ,\xi \right)\dot{\theta }+\varkappa \left(\sigma ,\xi \right)\dot{\sigma }-\dot{\xi }+\lambda \left(\theta \right)\left[\alpha \xi -S\left(\theta \right)+\Sigma \left(\sigma \right)\right] & \\ +g\left(\xi \right)\;\mathrm{sgn}\left[\alpha \xi -S\left(\theta \right)+\Sigma \left(\sigma \right)\right]\;[\beta \left(\theta ,\xi \right)|\dot{\theta }|+\nu \left(\sigma ,\xi \right)|\dot{\sigma }|] & =0. \end{array}$$

The constraint ξ∈[0,1] suggests that we assume

(30) $$\kappa \left(\theta ,\xi \right),\beta \left(\theta ,\xi \right),\varkappa \left(\sigma ,\xi \right),\nu \left(\sigma ,\xi \right)\to 0\;\mathrm{as}\;\xi \to 0_{+},1_{-}.$$

This assumption will be discussed in detail in the following Proposition 1. Hence, the CD (in)equality (29) results in a first-order, non-linear, differential equation for the evolution of the three functions θ(t),σ(t),ξ(t).

Next developments are mainly devoted to the evolutions at constant stress or at constant temperature to highlight the dynamics of temperature-induced or stress-induced transitions, respectively. Notice that when both temperature and stress are constant, $\theta =\theta_{c},\sigma =\sigma_{c}$, then $\dot{\theta }=0,\dot{\sigma }=0$ and Equation (31) reduces to

$$\dot{\xi }=\lambda \left(\theta \right)\left[\alpha \xi -S\left(\theta \right)+\Sigma \left(\sigma \right)\right],$$

which describes the purely diffusive aspect of the transition under steady-state thermomechanical conditions. This aspect is certainly relevant in transition models describing melting and solidification phenomena where the time derivative of the interested variable is given by the state variables and the possible spatial derivatives (see, e.g., [25,26,27]). Since experimental data show that ξ undergoes rate-independent hysteretic effects when the evolution is induced by time dependencies of temperature or stress, macroscopic models of SMAs are most often given by relations among time rates of temperature and stress (see, e.g., [17], [15] chapter 3). We pursue this view and then, for the present purposes, we assume λ=0. Hence, hereafter, Equation (29) is restricted to

(31) $$-\kappa \left(\theta ,\xi \right)\dot{\theta }+\varkappa \left(\sigma ,\xi \right)\dot{\sigma }-\dot{\xi }+g\left(\xi \right)\;\mathrm{sgn}\left[\alpha \xi -S\left(\theta \right)+\Sigma \left(\sigma \right)\right]\;[\beta \left(\theta ,\xi \right)|\dot{\theta }\left|+\nu \left(\sigma ,\xi \right)\right|\dot{\sigma }\left|\right]=0.$$

The restriction to evolutions induced by a varying temperature or stress allows us to clarify the corresponding properties of processes and possibly cycles. The model of the pseudoelastic behavior of a shape memory alloy is recovered as follows. At constant temperature, stress-strain curves are obtained from (24) with ξ obtained by integration of (31) over loading-unloading processes. If J=J(θ,σ) then (24) is formally modified by stating that δ is ${\left(1+\sigma \partial_{\sigma }J/J\right)}^{-1}$ times the right-hand side of the present (24) while everything else is left unchanged.

### 5.5. Transition Processes

At constant stress, say $\sigma =\sigma_{c}$, it is $\dot{\sigma }=0$ and Equation (31) simplifies to

(32) $$\dot{\xi }=-\kappa \left(\theta ,\xi \right)\dot{\theta }+\beta \left(\theta ,\xi \right)g\left(\xi \right)\;\mathrm{sgn}\left[\alpha \xi -S\left(\theta \right)+\Sigma \left(\sigma_{c}\right)\right]\left|\dot{\theta }\right|.$$

Instead, at constant temperature, say $\theta =\theta_{c}$, it is $\dot{\theta }=0$ and Equation (31) simplifies to

(33) $$\dot{\xi }=\varkappa \left(\sigma ,\xi \right)\dot{\sigma }+\nu \left(\sigma ,\xi \right)g\left(\xi \right)\;\mathrm{sgn}\left[\alpha \xi -S\left(\theta_{c}\right)+\Sigma \left(\sigma \right)\right]\left|\dot{\sigma }\right|.$$

For definiteness we let Equations (32) and (33) hold as t≥0 with given initial values $\theta \left(0\right)=\theta_{0},\sigma \left(0\right)=\sigma_{0}$.

In addition to the limit conditions (30), for the sake of simplicity the functions β and ν are taken to be equal to κ and ϰ, respectively, namely

(34) β(θ,ξ):=κ(θ,ξ), ν(σ,ξ):=ϰ(σ,ξ).

Moreover we put

(35) $$g\left(\xi \right)=\{\begin{array}{cc} 1 & \mathrm{if}\;0\leq \xi \leq 1,\\ -1 & \mathrm{otherwise}. \end{array}$$

We can then prove the following

**Proposition** **1.** *Under the assumptions (30), (34) and (35) the evolution Equations (32) and (33) yield trajectories that satisfy the constraint ξ(t)∈[0,1] for all t≥0.*

**Proof.**  The functions κ,ϰ and β,ν are strictly positive when $\left(\theta ,\sigma ,\xi \right)\in R^{+}\times R^{+}\times \left(0,1\right)$ and satisfy the conditions (30) and (34). For generality, we distinguish two cases according as κ,ϰ tend to zero asymptotically while $\xi \to 0_{+},1_{-}$, or keep the null value at ξ=0,1, namely

(36) $$\lim_{\xi \to 0^{+}}\kappa \left(\theta ,\xi \right)=\lim_{\xi \to 1_{-}}\kappa \left(\theta ,\xi \right)=0,\;\;\;\;\lim_{\xi \to 0_{+}}\varkappa \left(\sigma ,\xi \right)=\lim_{\xi \to 1_{-}}\varkappa \left(\sigma ,\xi \right)=0,$$

or

(37) κ(θ,0)=κ(θ,1)=0,    ϰ(σ,0)=ϰ(σ,1)=0,

for all θ,σ>0.

We start with the evolution at constant stress and then apply the evolution Equation (32) as t≥0 and the initial values $\xi \left(0\right)=\xi_{0},\theta \left(0\right)=\theta_{0}$ with $\theta_{0}$ small enough so that $\alpha \xi_{0}-S\left(\theta_{0}\right)+\Sigma \left(\sigma_{c}\right)>0$. Let $\dot{\theta }>0$. Owing to the assumption κ(θ,ξ)=β(θ,ξ) we can write Equation (32) in the form

(38) $$\dot{\xi }=-\kappa \left(\theta ,\xi \right)\left\{1-g\left(\xi \right)\;\mathrm{sgn}\left[\alpha \xi -S\left(\theta \right)+\Sigma \left(\sigma_{c}\right)\right]\right\}\dot{\theta }.$$

**Figure 3:**
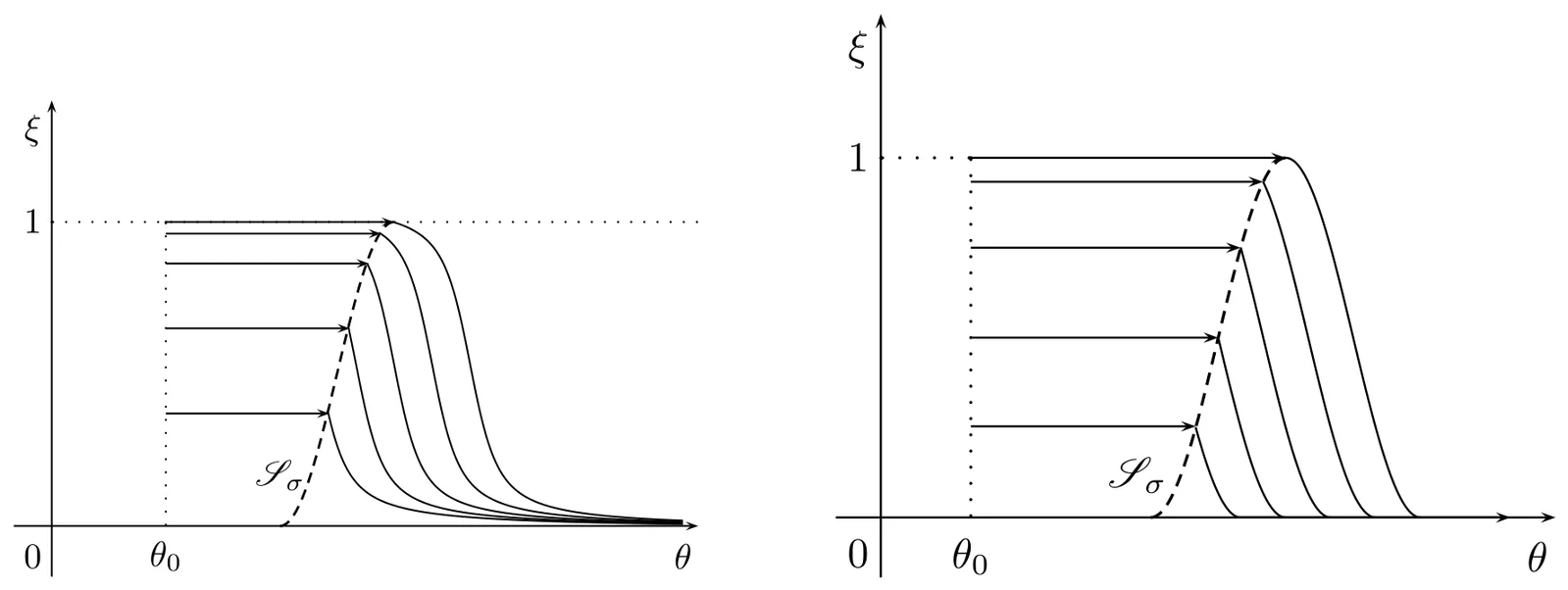
The figure shows the dependence of the martensite fraction ξ as the temperature θ increases in the case κ satisfies the condition (36) (**left**) or (37) (**right**).

Hence

$$\dot{\xi }=-\kappa \left(\theta ,\xi \right)\left[1-g\left(\xi \right)\right]\dot{\theta }$$

shows that, as t≥0, it is $\dot{\xi }=0$. Since θ increases then S(θ) too increases. Accordingly, there can be a time $t^{*}$ such that $\alpha \xi_{0}=S\left(\theta \left(t^{*}\right)\right)-\Sigma \left(\sigma_{c}\right)$. As $t>t^{*}$ and θ still increases it follows $\dot{\xi }=-2\kappa \left(\theta ,\xi \right)\dot{\theta }$ whence ξ tends asymptotically to 0, or attains 0 in a finite time, depending on the validity of (36) or (37), as θ increases indefinitely (see Figure 3).

**Figure 4:**
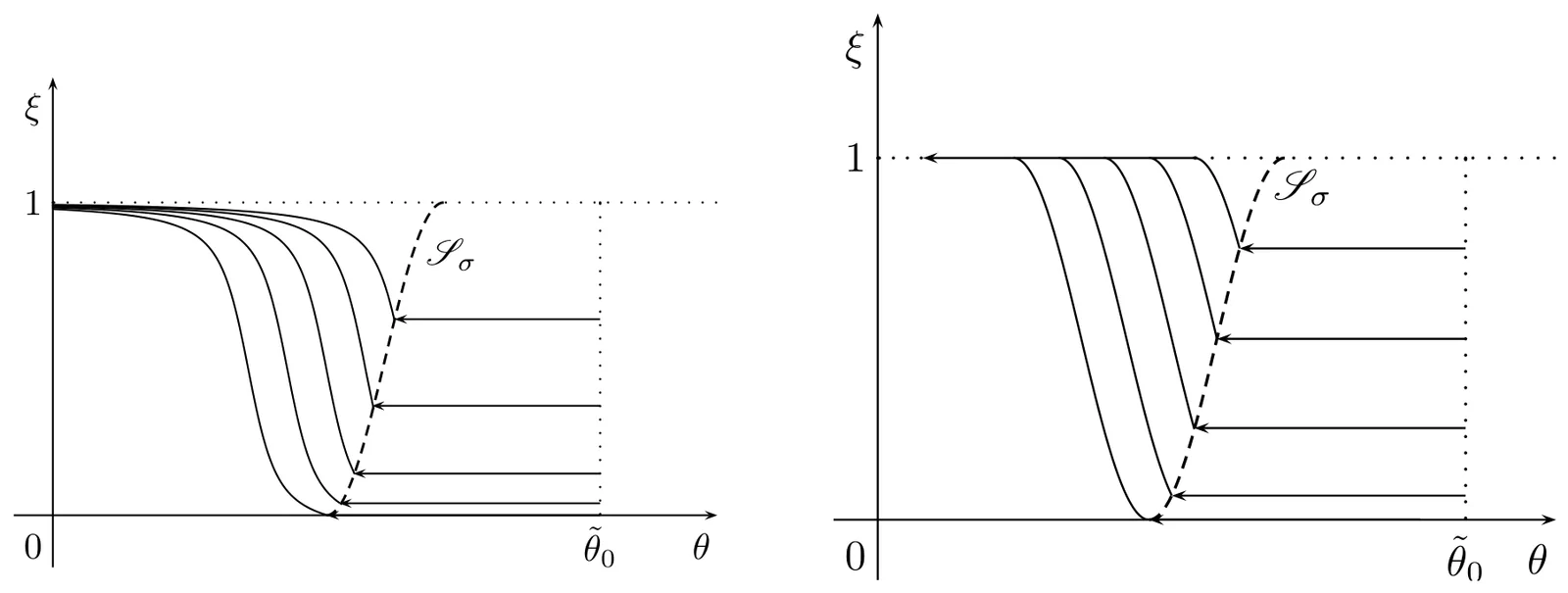
The figure shows the dependence of the martensite fraction ξ as the temperature θ increases in the case κ satisfies the condition (36) (**left**) or (37) (**right**).

Instead, let now $\dot{\theta }<0$ and $\xi \left(0\right)=\xi_{0},\theta \left(0\right)={\tilde{\theta }}_{0}$. The initial value $\alpha \xi_{0}$ is sufficiently large so that $\alpha \xi_{0}-S({\tilde{\theta }}_{0}-\Sigma \left(\sigma_{c}\right)<0$. Consequently, the evolution Equation (32) becomes

$$\dot{\xi }=-\kappa \left(\theta ,\xi \right)\left[1-g\left(\xi \right)\right]\dot{\theta }=0$$

inasmuch as $\alpha \xi_{0}-S\left(\theta \left(t\right)\right)+\Sigma \left(\sigma_{c}\right)<0$. Hence $\xi =\xi_{0}$ up to a time $\bar{t}$ and temperature $\bar{\theta }=\theta \left(\bar{t}\right)$ such that $\alpha \xi_{0}-S\left(\bar{\theta }\right)+\Sigma \left(\sigma_{c}\right)=0$. As $t>\bar{t}$ we have $\alpha \xi_{0}-S\left(\theta \left(t\right)\right)+\Sigma \left(\sigma_{c}\right)>0$ and then $\dot{\xi }=-2\kappa \dot{\theta }>0$; the value of ξ increases up to the asymptotic limit ξ=1 or ξ attains 1 in a finite time depending on the validity of (36) or (37) (see Figure 4).

**Figure 5:**
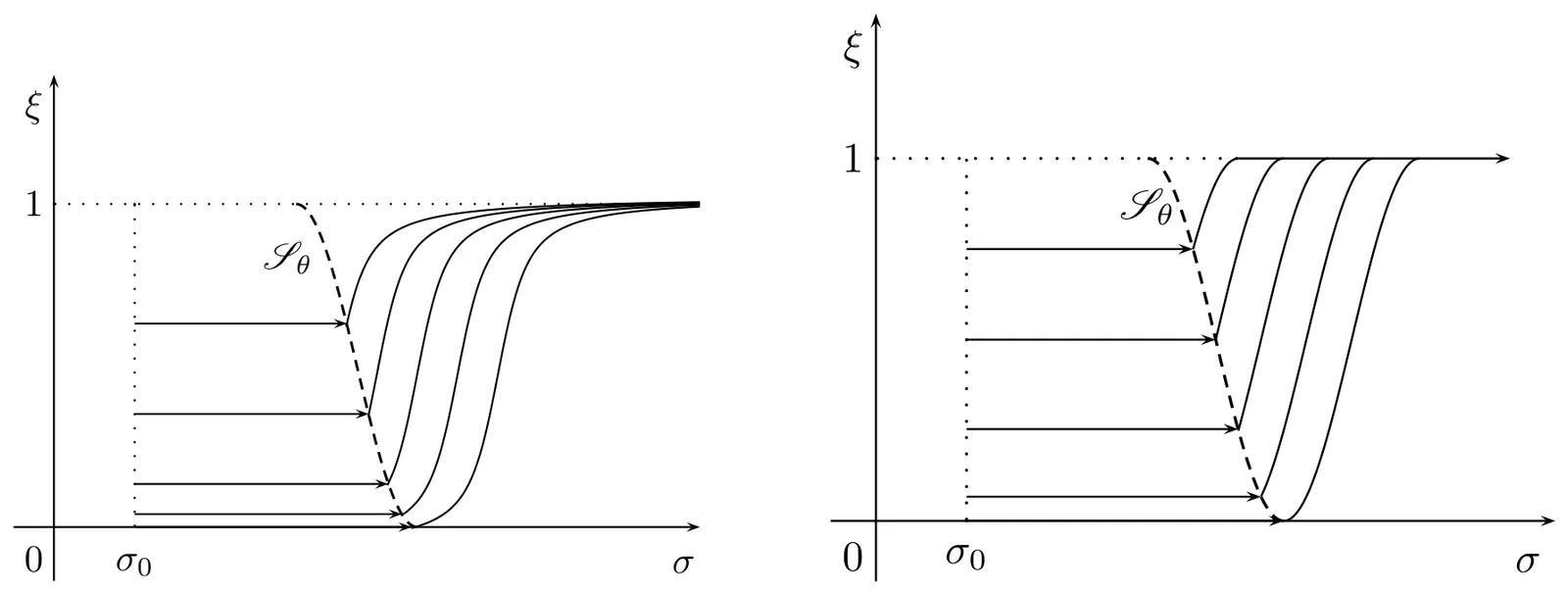
Dependence of the martensite fraction ξ as the stress σ increases in the case ϰ satisfies the condition (36) (**left**) or (37) (**right**).

**Figure 6:**
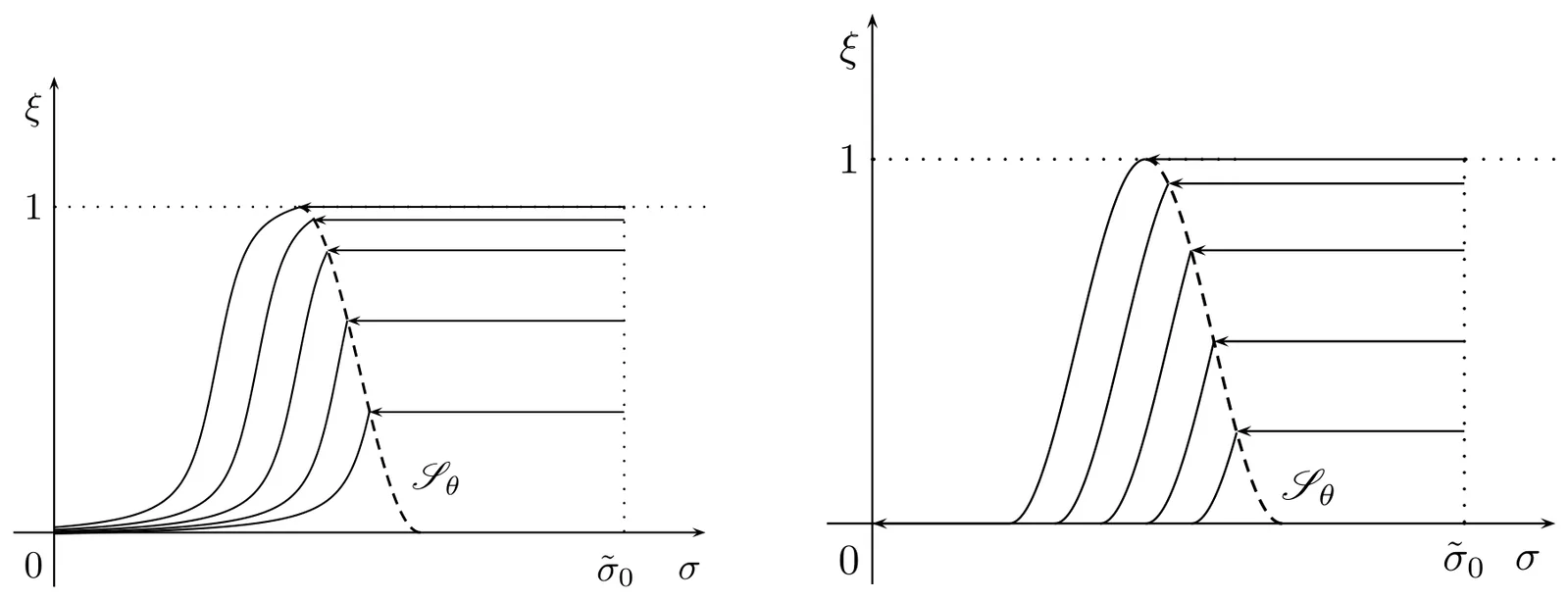
Dependence of the martensite fraction ξ as the stress σ decreases in the case ϰ satisfies the condition (36) (**left**) or (37) (**right**).

The proof that ξ→1 if $\dot{\sigma }>0$ and ξ→0 if $\dot{\sigma }<0$ follows in the same way through the evolution Equation (33); cf. Figure 5 and Figure 6. □

The pair of Figure 3, Figure 4, Figure 5 and Figure 6 show the different meaning of the assumptions (36) and (37). The assumption (36) implies that the transition happens asymptotically, with the saturation property, where the zero slope is achieved as $\xi \to 0_{+}$ or $1_{-}$ (see figures on the left). Instead, the assumption (37) implies that austenite (ξ=0) and martensite (ξ=1) are obtained at a finite time, with zero slope (see figures on the right). In Figure 3, Figure 4, Figure 5 and Figure 6 we used Langevin-shaped or cosine-shaped curves to satisfy the conditions (36) or (37), respectively. In both cases, the coefficients κ and ϰ are functions of the variable ξ alone. For example, in the case of the figures on the right we set $\kappa \left(\xi \right)=\varkappa \left(\xi \right)=\sqrt{1-{\left(2\xi -1\right)}^{2}}$.

**Figure 7:**
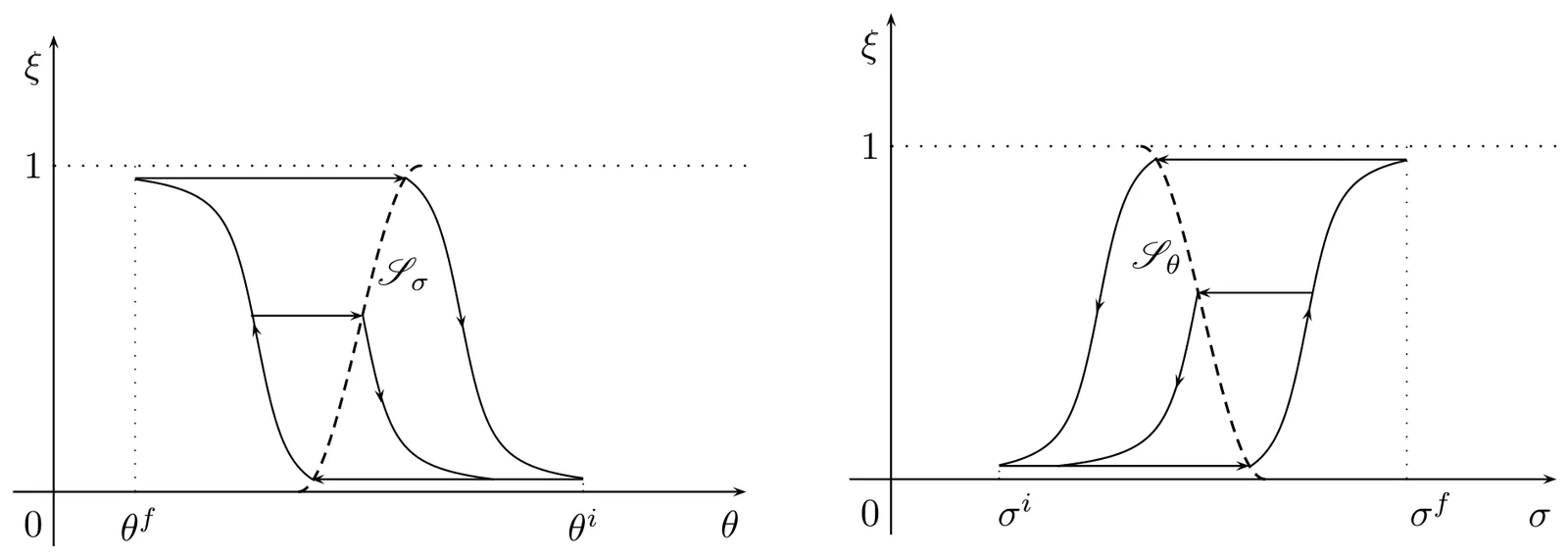
Major and minor loops can arise in the θ−ξ plane (**left**) and in the σ−ξ plane (**right**) if the condition (36) for κ and ϰ is satisfied.

Figure 7 shows the evolution of ξ when the condition (36) is satisfied whence ξ=0 or ξ=1 can be obtained asymptotically. On the left, the loop describes a closed temperature cycle at constant stress. On the right, the loop describes a closed stress cycle at constant temperature. Both loops follow the properties of the model.

At constant stress, $\sigma =\sigma_{C}$, let the starting point be at $\theta =\theta^{i}$ on the right of $S_{\sigma }$. Next, θ can decrease while $\xi =\xi^{i}$, thus following a horizontal trajectory, until $S_{\sigma }$ is reached. As θ keeps decreasing the mass fraction ξ increases and reaches the value $\xi^{f}<1$ at the temperature $\theta^{f}<\theta^{i}$. If now the temperature increases then ξ keeps constant, $\xi =\xi^{f}$, until the trajectory reaches the equilibrium line $S_{\sigma }$. Next, if the temperature increases, then ξ decreases. If the trajectory reaches the point $\left(\theta^{i},\xi^{i}\right)$, then the whole trajectory is a major (closed) loop (see Figure 7 left).

Likewise, as θ is constant, say $\theta =\theta_{C}$, a loop follows that is run in the counterclockwise sense (see Figure 7 right).

**Figure 8:**
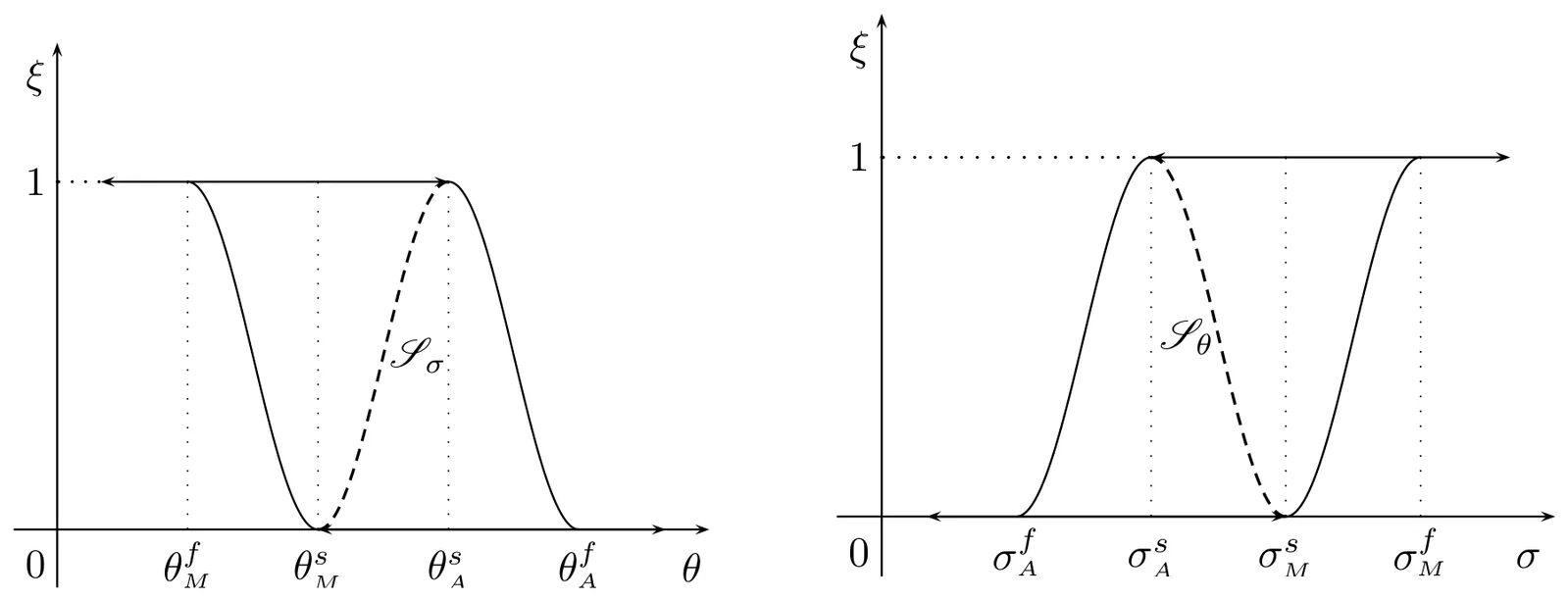
Major loops can arise in the θ−ξ plane (**left**) and in the σ−ξ plane (**right**) if the condition (37) for κ and ϰ is satisfied.

The condition (37), for κ and ϰ, implies that the limit values ξ=0 and ξ=1 are reached at finite values of σ and θ. Consequently, major loops are easier to follow. First let the stress be constant, $\sigma =\sigma_{C}$ and consider a starting point with ξ=0 and $\theta =\theta^{i}>\theta_{A}^{f}$ (see Figure 8 left). As θ decreases, the trajectory is horizontal (ξ=0) until the curve S is reached when $\theta =\theta_{M}^{s}$. If the temperature keeps decreasing, then ξ increases up to ξ=1 in a finite time at $\theta =\theta_{M}^{f}$. The reverse process holds by starting from ξ=1 and $\theta \leq \theta_{M}^{f}$ and increasing the temperature. The final state is obtained with ξ=0 and $\theta =\theta_{A}^{f}$. An analogous evolution, induced by the stress, follows with a constant temperature (see Figure 8, right).

## 6. Non-Local Models for SMAs

As a generalization of the modeling of SMAs, we may allow for the dependence of the alloy on non-uniform fields. Accordingly, we now look for a thermodynamic scheme where the temperature θ, the mass fraction ξ, and the traction σ depend on the position and the constitutive functions are affected by their gradients. Here we show how two schemes can be established in Lagrangian and Eulerian descriptions.

### 6.1. Lagrangian Formulation

Consider the referential vectors

$$k_{R}=JkF^{-T},\;q_{R}=JqF^{-T}.$$

Since (see, e.g., [16], § 2.5)

$$J\nabla \cdot k=\nabla_{\;\;R}\cdot k_{R},\;Jq\cdot \nabla \theta =q_{R}\cdot \nabla_{\;\;R}\theta$$

then we can write the CD inequality (7) in the form

(39) $$-\rho_{R}\left(\dot{\phi }+\tilde{\eta }\dot{\theta }\right)-\delta J\dot{\sigma }+\theta \nabla_{\;\;R}\cdot k_{R}-\frac{1}{\theta }q_{R}\cdot \nabla_{\;\;R}\theta =\rho_{R}\theta \gamma .$$

We generalize the thermodynamic scheme of Section 3 by letting

$$\theta ,\xi ,\sigma ,\nabla_{\;\;R}\theta ,\nabla_{\;\;R}\xi ,\nabla_{\;\;R}\sigma ,\dot{\theta },\dot{\sigma }$$

be the variables and

$$\phi ,{\tilde{\eta }}^{dyn},\delta^{dyn},k_{R},q_{R},\gamma$$

be the constitutive functions. Upon computation of $\dot{\phi }$ and substitution in (39) we obtain

$$\begin{array}{c} -\rho_{R}\left(\partial_{\theta }\phi +\tilde{\eta }\right)\dot{\theta }-\rho_{R}\left(\partial_{\sigma }\phi +\delta /\rho \right)\dot{\sigma }-\rho_{R}\partial_{\nabla_{\;\;R}\theta }\phi \cdot \nabla_{\;\;R}\dot{\theta }-\rho_{R}\partial_{\nabla_{\;\;R}\xi }\phi \cdot \nabla_{\;\;R}\dot{\xi }\;\;\\ -\rho_{R}\partial_{\nabla_{\;\;R}\sigma }\phi \cdot \nabla_{\;\;R}\dot{\sigma }-\rho_{R}\partial_{\xi }\phi \;\dot{\xi }-\rho_{R}\partial_{\dot{\theta }}\phi \cdot \ddot{\theta }-\rho_{R}\partial_{\dot{\sigma }}\phi \cdot \ddot{\sigma }+\theta \nabla_{\;\;R}\cdot k_{R}-\frac{1}{\theta }q_{R}\cdot \nabla_{\;\;R}\theta =\rho_{R}\theta \gamma . \end{array}$$

The linearity and arbitrariness of $\ddot{\theta }$ and $\ddot{\sigma }$ imply that

$$\partial_{\dot{\theta }}\phi =0,\;\partial_{\dot{\sigma }}\phi =0.$$

We now divide the remaining inequality by θ to find

(40) $$\begin{array}{c} -\frac{\rho_{R}}{\theta }\left(\partial_{\theta }\phi +\tilde{\eta }\right)\dot{\theta }-\frac{\rho_{R}}{\theta }\left(\partial_{\sigma }\phi +\delta /\rho \right)\dot{\sigma }-\frac{\rho_{R}}{\theta }\partial_{\nabla_{\;\;R}\theta }\phi \cdot \nabla_{\;\;R}\dot{\theta }-\frac{\rho_{R}}{\theta }\partial_{\nabla_{\;\;R}\xi }\phi \cdot \nabla_{\;\;R}\dot{\xi }\;\;\\ -\frac{\rho_{R}}{\theta }\partial_{\nabla_{\;\;R}\sigma }\phi \cdot \nabla_{\;\;R}\dot{\sigma }-\frac{\rho_{R}}{\theta }\partial_{\xi }\phi \;\dot{\xi }+\nabla_{\;\;R}\cdot k_{R}-\frac{1}{\theta^{2}}q_{R}\cdot \nabla_{\;\;R}\theta =\rho_{R}\gamma . \end{array}$$

Next, we apply the identity

$$-\frac{\rho_{R}}{\theta }\partial_{\nabla_{\;\;R}\xi }\phi \cdot \nabla_{\;\;R}\dot{\xi }=-\nabla_{\;\;R}\cdot \left(\frac{\rho_{R}}{\theta }\partial_{\nabla_{\;\;R}\xi }\phi \;\dot{\xi }\right)+\left[\nabla_{\;\;R}\cdot \left(\frac{\rho_{R}}{\theta }\partial_{\nabla_{\;\;R}\xi }\phi \right)\right]\dot{\xi }$$

and the analogous ones for $\nabla_{\;\;R}\dot{\theta },\nabla_{\;\;R}\dot{\sigma }$. We can then write (40) in the form

(41) $$\begin{array}{c} -\frac{\rho_{R}}{\theta }\left(D_{\theta }\phi +\tilde{\eta }\right)\dot{\theta }-\frac{\rho_{R}}{\theta }D_{\xi }\phi \;\dot{\xi }-\frac{\rho_{R}}{\theta }\left(D_{\sigma }\phi +\delta /\rho \right)\dot{\sigma }-\frac{1}{\theta^{2}}q_{R}\cdot \nabla_{\;\;R}\theta \;\;\\ +\nabla_{\;\;R}\cdot \left[k_{R}-\frac{\rho_{R}}{\theta }\partial_{\nabla_{\;\;R}\theta }\phi \;\dot{\theta }-\frac{\rho_{R}}{\theta }\partial_{\nabla_{\;\;R}\xi }\phi \;\dot{\xi }-\frac{\rho_{R}}{\theta }\partial_{\nabla_{\;\;R}\sigma }\phi \;\dot{\sigma }\right]=\rho_{R}\gamma . \end{array}$$

where

(42) $$D_{\xi }\;\phi =\partial_{\xi }\phi -\frac{\theta }{\rho_{R}}\nabla_{\;\;R}\cdot \left(\frac{\rho_{R}}{\theta }\partial_{\nabla_{\;\;R}\xi }\phi \right),$$

and the analog holds for $D_{\theta },D_{\sigma }$. Consequently we let

$$k_{R}=\frac{\rho_{R}}{\theta }\partial_{\nabla_{\;\;R}\theta }\phi \;\dot{\theta }+\frac{\rho_{R}}{\theta }\partial_{\nabla_{\;\;R}\xi }\phi \;\dot{\xi }+\frac{\rho_{R}}{\theta }\partial_{\nabla_{\;\;R}\sigma }\phi \;\dot{\sigma }$$

and then it follows

(43) $$-\left(D_{\theta }\phi +\tilde{\eta }\right)\dot{\theta }-D_{\xi }\phi \;\dot{\xi }-\left(D_{\sigma }\phi +\delta /\rho \right)\dot{\sigma }-\frac{1}{\theta^{2}}q_{R}\cdot \nabla_{\;\;R}\theta =\rho_{R}\gamma .$$

We can view the definition (42) as a generalized form of the variational derivative of ϕ with respect to ξ. Indeed, if $\rho_{R}$ and θ are constant then

$$D_{\xi }\phi =\partial_{\xi }\phi -\nabla_{\;\;R}\cdot \partial_{\nabla_{\;\;R}\xi }\phi ,$$

or, in one dimension,

(44) $$D_{\xi }\phi =\partial_{\xi }\phi -\partial_{X}\partial_{\partial_{X}\xi }\phi .$$

The expression (44) is the classical form of variational derivatives in mathematical analysis (see, e.g., [28]).

Though ϕ is allowed to depend on $\nabla_{\;\;R}\theta$ we assume that

$$D_{\theta }\phi +\tilde{\eta },\;D_{\xi }\phi ,\;D_{\sigma }\phi +\delta /\rho$$

and $\dot{\xi }$ are independent of $\nabla_{\;\;R}\theta$. Thus Equation (43) implies that

(45) $$-\left(D_{\theta }\phi +\tilde{\eta }\right)\dot{\theta }-D_{\xi }\phi \;\dot{\xi }-\left(D_{\sigma }\phi +\delta /\rho \right)\dot{\sigma }=\rho_{R}\gamma_{0},$$

where $\gamma_{0}$ is the non-negative value of γ as $\nabla_{\;\;R}\theta =0$. Let

$${\tilde{\eta }}^{dyn}:=D_{\theta }\phi +\tilde{\eta },\;\delta^{dyn}=\rho D_{\sigma }\phi +\delta$$

whence

$$\tilde{\eta }:=-D_{\theta }\phi +{\tilde{\eta }}^{dyn},\;\delta =-\rho D_{\sigma }\phi +\delta^{dyn}.$$

Hence the CD inequality (45) can be written in the form

(46) $$-{\tilde{\eta }}^{dyn}\dot{\theta }-D_{\xi }\phi \;\dot{\xi }-\frac{1}{\rho }\delta^{dyn}\dot{\sigma }=\rho_{R}\gamma_{0}.$$

If $D_{\xi }\phi \neq 0$ then Equation (46) can be viewed as the evolution equation for ξ(X,t). Accordingly, we can view

(47) $$D_{\xi }\phi =0$$

as the equilibrium equation for the functions ξ(X),θ(X),σ(X).

As happens, in general, for non-local models (see, e.g., [8,9]), a non-zero extra-entropy flux k occurs. The expression of k is found in terms of the free energy ϕ. Since the terms of k are associated with the variational derivatives of ϕ, the validity of the whole model is ascertained by the validity of (43) and hence through (46). The next step is to relate $\delta^{dyn}$ and ${\tilde{\eta }}^{dyn}$ to ϕ in the form

$${\tilde{\eta }}^{dyn}=\kappa \left(\theta ,\xi \right)D_{\xi }\phi ,\;\delta^{dyn}=-\rho \varkappa \left(\sigma ,\xi \right)D_{\xi }\phi .$$

The equilibrium condition (47) has the form of the Euler–Lagrange equation arising from the minimum of the Landau–Ginzburg functional [29,30]. The evolution equation for the modeling of phase transitions is derived in [29] by requiring the decrease in free energy along solutions ξ(X,t), and in [30] by requiring the relaxation toward equilibrium, viz.

(48) $$\beta \dot{\xi }=-D_{\xi }\phi .$$

If $\dot{\theta }=0$ and $\dot{\sigma }=0$ then Equation (46) simplifies to

(49) $$D_{\xi }\phi \;\dot{\xi }=-\rho_{R}\gamma_{0}$$

and the evolution Equation (48) is (thermodynamically) consistent with (49) subject to $\gamma_{0}={\left(D_{\xi }\phi \right)}^{2}/\rho_{R}\beta$. Accordingly, we conclude that equilibrium holds with zero entropy production.

As an example of (47) we let

$$\phi =F\left(\xi \right)+\xi \left[g_{1}\left(\theta \right)+g_{2}\left(\sigma \right)\right]+h\left(\theta ,\sigma \right)+\frac{1}{2}\alpha {\left|\nabla_{\;\;R}\xi \right|}^{2},$$

where $g_{1},g_{2}$ and *h* are independent of ξ and $\nabla_{\;\;R}\xi$. If θ and σ are constant then $g=g_{1}+g_{2}$ and *h* are constant and the equilibrium Equation (47) takes the form

(50) $$f\left(\xi \right)+g-\alpha \partial_{X}^{2}\xi =0,$$

where $f\left(\xi \right)=F^{\prime }\left(\xi \right)$. Upon multiplying Equation (50) by $\partial_{X}\xi$ we find

$$\partial_{X}\left[-\frac{\alpha }{2}{\left(\partial_{X}\xi \right)}^{2}+F\left(\xi \right)+g\xi \right]=0.$$

The constancy of $-\frac{\alpha }{2}{\left(\partial_{X}\xi \right)}^{2}+F\left(\xi \right)+g\xi$ with respect to *X* results in the first-order differential equation

$$-\frac{\alpha }{2}{\left(\partial_{X}\xi \right)}^{2}+F\left(\xi \right)+g\xi =c$$

for the spatial dependence of ξ at each time *t*.

We observe that $\partial_{X}=F\partial_{x}$ and then $\partial_{X}≃\partial_{x}$ inasmuch as F≃1.

### 6.2. Eulerian Formulation

A direct Eulerian formulation involves the gradients ∇ξ,∇θ. Owing to the occurrence of the velocity gradient L in the identity

$$\dot{\left(\nabla \xi \right)}=\nabla \dot{\xi }-L^{T}\nabla \xi ,$$

and the like for θ, we overcome some technical difficulties by simplifying the model of the body. Consistent with the uniaxial geometry of the body, we let θ,ξ,v depend on the current position x through the component *x* in the longitudinal direction $e_{1}$. Since $L_{11}=\dot{F}F^{-1}=\dot{\delta }$ then it follows that

$$\nabla \xi =\partial_{x}\xi \;e_{1},\;\dot{\left(\partial_{x}\xi \right)}=\partial_{x}\dot{\xi }-\dot{\delta }\;\partial_{x}\xi .$$

The CD inequality (6) may then be written in the one-dimensional form

(51) $$-\rho \left(\dot{\psi }+\eta \dot{\theta }\right)+\sigma \dot{\delta }+\theta \partial_{x}k-\frac{1}{\theta }q\;\partial_{x}\theta =\rho \theta \gamma ,$$

where $k=k\cdot e_{1}$. Let

$$\theta ,\xi ,\delta ,\partial_{x}\theta ,\partial_{x}\xi ,\dot{\theta }$$

be the variables and

$$\eta ,\sigma ,\dot{\xi },k,q,\gamma$$

the constitutive functions. For simplicity, we assume ψ is independent of $\partial_{x}\theta$. Hence upon computation of $\dot{\psi }$ and substitution in (51) we have

$$-\rho \left(\partial_{\theta }\psi +\eta \right)\dot{\theta }-\rho \partial_{\xi }\psi \;\dot{\xi }-\rho \partial_{\partial_{x}\xi }\psi \dot{\left(\partial_{x}\xi \right)}+\left(-\rho \partial_{\delta }\psi +\sigma \right)\dot{\delta }-\rho \partial_{\dot{\theta }}\psi \ddot{\theta }+\theta \partial_{x}k-\frac{1}{\theta }q\partial_{x}\theta =\rho \theta \gamma .$$

The linearity and arbitrariness of $\ddot{\theta }$ imply that

$$\partial_{\dot{\theta }}\psi =0.$$

We now divide the remaining equation by θ and notice that

$$\begin{array}{cc} -\frac{\rho }{\theta }\partial_{\partial_{x}\xi }\psi \dot{\left(\partial_{x}\xi \right)} & =\frac{\rho }{\theta }\partial_{\partial_{x}\xi }\psi \;\partial_{x}\dot{\xi }-\frac{\rho }{\theta }\partial_{\partial_{x}\xi }\psi \partial_{x}\xi \;\dot{\delta }\\ & =\partial_{x}\left(\frac{\rho }{\theta }\partial_{\partial_{x}\xi }\psi \;\dot{\xi }\right)-\left[\partial_{x}\left(\frac{\rho }{\theta }\partial_{\partial_{x}\xi }\psi \right)\right]\dot{\xi }-\frac{\rho }{\theta }\partial_{\partial_{x}\xi }\psi \;\partial_{x}\xi \;\dot{\delta }. \end{array}$$

Hence we have

(52) $$\begin{array}{c} -\frac{\rho }{\theta }\left(\partial_{\theta }\psi +\eta \right)\dot{\theta }-\frac{\rho }{\theta }\left(\partial_{\delta }\psi -\sigma /\rho +\partial_{x}\xi \partial_{\partial_{x}\xi }\psi \right)\dot{\delta }-\frac{\rho }{\theta }D_{\xi }\psi \dot{\xi }\\ +\partial_{x}\left(k-\frac{\rho }{\theta }\partial_{\partial_{x}\xi }\psi \dot{\xi }\right)-\frac{1}{\theta^{2}}q\partial_{x}\theta =\rho \gamma , \end{array}$$

where

$$D_{\xi }\psi =\partial_{\delta }\psi -\frac{\theta }{\rho }\partial_{x}\left(\frac{\rho }{\theta }\partial_{\partial_{x}\xi }\psi \right).$$

Hence we let

$$k=\frac{\rho }{\theta }\partial_{\partial_{x}\xi }\psi \dot{\xi }.$$

Define

$$\eta^{dyn}=\eta +\partial_{\theta }\psi ,\;\sigma^{dyn}=\sigma -\rho \left(\partial_{\delta }\psi +\partial_{x}\xi \partial_{\partial_{x}\xi }\psi \right)$$

whence

(53) $$\eta =-\partial_{\theta }\psi +\eta^{dyn},$$

(54) $$\sigma =\rho (\partial_{\delta }\psi +\left(\partial_{x}\xi \partial_{\partial_{x}\xi }\psi \right)+\sigma^{dyn}.$$

Accordingly, η and σ are determined as soon as the functions $\eta^{dyn},\sigma^{dyn}$, and the free energy ψ are specified. This step is accomplished in a moment.

An additive decomposition of η and σ holds in the form (8) provided we let

$$\eta^{el}=-\partial_{\theta }\psi ,\;\sigma^{el}=\rho \left(\partial_{\delta }\psi +\partial_{x}\xi \;\partial_{\partial_{x}\xi }\psi \right)$$

thus regarding $\eta^{el},\sigma^{el}$ as the elastic part of η and σ.

If $\eta^{dyn},\sigma^{dyn}$, and $D_{\xi }\psi \;\dot{\xi }$ are independent of $\partial_{x}\theta$ then the CD inequality (52) reduces to

(55) $$-\rho \eta^{dyn}\dot{\theta }+\sigma^{dyn}\dot{\delta }-\rho D_{\xi }\psi \;\dot{\xi }=\rho \theta \gamma_{0}$$

where $\gamma_{0}$ is the (non-negative) value of γ when $\partial_{x}\theta =0$. In accordance with (55), the functions $\eta^{dyn}$ and $\sigma^{dyn}$ describe the dynamics of the alloy. Now, if $\eta^{dyn}$ and $\sigma^{dyn}$ are zero, then the equilibrium of the system is characterized by the vanishing of $D_{\xi }\psi$. Accordingly, we extend the equilibrium property of $D_{\xi }\psi =0$ by assuming that

(56) $$\eta^{dyn}=\varphi \left(\theta ,\xi \right)D_{\xi }\psi ,\;\;\;\;\sigma^{dyn}=\rho \omega \left(\delta ,\xi \right)D_{\xi }\psi ,\;\;\;\;\varphi ,\omega >0.$$

As to the entropy production $\gamma_{0}$, we account for the postulate of the second law by letting $\gamma_{0}$ be a positive-valued function of $D_{\xi }\psi ,\dot{\theta },\dot{\delta }$ in the form

$$\theta \gamma =\lambda \left(\theta \right)|D_{\xi }{\psi |}^{2}+g\left(\xi \right)\;|D_{\xi }\psi |(\beta |\dot{\theta }\left|+\mu \right|\dot{\delta }\left|\right),$$

where g(ξ)>0 as ξ∈[0,1] and λ,β,μ are non-negative functions. Incidentally, it is $\gamma =\gamma_{0}$.

In accordance with (56), the functions $\eta^{dyn},\sigma^{dyn}$ describe the dynamics of the alloy. Indeed, if $\eta^{dyn}$ and $\sigma^{dyn}$ are zero, then the equilibrium of the system is characterized by the vanishing of $D_{\xi }\psi$. Accordingly, we extend the equilibrium property of $D_{\xi }\psi =0$ by assuming that

(57) $$\eta^{dyn}=\varphi \left(\theta ,\xi \right)D_{\xi }\psi ,\;\;\;\;\sigma^{dyn}=\rho \omega \left(\delta ,\xi \right)D_{\xi }\psi ,\;\;\;\;\varphi ,\omega >0,$$

where the functions φ and ω are independent of the free energy ψ.

Thus the evolution Equation (55) takes the form

(58) $$-\varphi \left(\theta ,\xi \right)\dot{\theta }+\omega \left(\delta ,\xi \right)\dot{\delta }-\dot{\xi }=\lambda \left(\theta \right)D_{\xi }\psi +g\left(\xi \right)\;\mathrm{sgn}\left(D_{\xi }\psi \right)\left(\beta \;\right|\dot{\theta }\left|+\mu \;\right|\dot{\delta }\left|\right),$$

with ξ∈[0,1]. The evolution Equation (58) is a nonlinear partial differential equation involving first-order time derivatives of θ,δ,ξ and second-order spatial derivatives of ξ.

If the dependence on ∇ξ is neglected then the evolution Equation (58) reduces to a first-order nonlinear differential equation for θ,δ, and ξ,

(59) $$-\varphi \left(\theta ,\xi \right)\dot{\theta }+\omega \left(\delta ,\xi \right)\dot{\delta }-\dot{\xi }=\lambda \left(\theta \right)\partial_{\xi }\psi +g\left(\xi \right)\;\mathrm{sgn}\left(\partial_{\xi }\psi \right)\;(\beta |\dot{\theta }+\mu |\dot{\delta }\left|\right).$$

Particular linear cases of Equation (59) allow a closed-form solution as is shown in [12] with λ=0.

At constant temperature it is $\dot{\theta }=0$ and Equation (58) takes the form

$$\dot{\xi }=\omega \left(\delta ,\xi \right)\dot{\delta }-g\left(\xi \right)\;\mathrm{sgn}\left(D_{\xi }\psi \right)\mu \left|\dot{\delta }\right|-\lambda \left(\theta \right)D_{\xi }\psi .$$

Instead, at constant deformation it is $\dot{\delta }=0$ and the evolution equation simplifies to

$$\dot{\xi }=-k\left(\theta ,\xi \right)\dot{\theta }-g\left(\xi \right)\;\mathrm{sgn}\left(D_{\xi }\psi \right)\;\beta \left|\dot{\theta }\right|-\lambda \left(\theta \right)D_{\xi }\psi .$$

Furthermore, if $\dot{\delta }=0$ and $\dot{\theta }=0$ then the evolution equation reduces to

(60) $$\dot{\xi }=-\lambda \left(\theta \right)D_{\xi }\psi .$$

Equation (60) shows that if ξ is not an equilibrium value, namely $D_{\xi }\psi \neq 0$, then ξ evolves in time toward equilibrium at the rate $-\lambda \left(\theta \right)D_{\xi }\psi$. Indeed, if $\lambda \left(\theta \right)=\lambda_{0}\rho /\theta$ and we let Ψ=ρψ/θ then Equation (60) takes the form

$$\dot{\xi }=-\lambda_{0}D_{\xi }\Psi$$

where D denotes the standard variational derivative or the Euler–Lagrange differential operator. This model equation traces back to a variational approach by Landau and Ginzburg, first applied to superconductivity [31] and next extended to a variety of phase transition phenomena (see [27,32,33] and references therein), including materials with hysteresis [34].

The internal energy ε:=ψ+θη is determined by the free energy ψ and the dynamic entropy $\eta^{dyn}$, namely

$$\varepsilon =\psi +\theta \left(\eta^{dyn}-\partial_{\theta }\psi \right)=\psi -\theta \partial_{\theta }\psi +\theta \varphi \left(\theta ,\xi \right)D_{\xi }\psi .$$

## 7. Conclusions

The second law of thermodynamics is applied to the formulation of a thermodynamically-consistent model of SMAs. Based on previous works of our own about hysteretic models, the second law is considered in the general form where the entropy flux j=q/θ+k and the entropy production γ are given by constitutive functions to be determined depending on the material under consideration.

The main purpose of the paper is to establish a continuum model of SMAs. The conceptual steps are the determination of a free energy ϕ(θ,σ,ξ) and a function for the entropy production γ. By viewing the alloy as a solid mixture, we arrive at the expression of ϕ as in (21). Next we establish the equilibrium surface in the form

$$\partial_{\xi }\phi =0$$

and the evolution equations for the behavior of the alloy outside the equilibrium surface. In particular, we obtain the evolution of the mass fraction ξ induced by a varying temperature θ or a tensile stress σ (see Equations (32) and (33)). Details are given as the structure of major loops in the θ−ξ or σ−ξ planes for ξ(θ) and ξ(σ), depending on whether the curve converges asymptotically or in a finite time.

Next, attention is given to models where ∇ξ is among the variables of the model. This scheme is considered in both Lagrangian and Eulerian formulations with the free energy ψ=ε−θη. The conclusion is that results similar to the previous case are obtained for the equilibrium surface and the evolution equation (see Equation (58)). The mathematical difficulty is the occurrence of the variational derivative $D_{\xi }\psi$ in place of the partial derivative $\partial_{\xi }\psi$. This generalization to non-local formulations, involving gradients of temperature, stress, and mass fraction, is shown to hold by virtue of suitable expressions of the extra-entropy flux k.

## Data Availability

No new data have been created. The study did not report any data.

## Abbreviations

The following abbreviations are used in this manuscript:

CD	Clausius–Duhem
SMAs	shape memory alloys

## References

[1] Truesdell C. (1984). Rational Thermodynamics.

[2] Coleman B.D., Noll W. (1963). The thermodynamics of elastic materials with heat conduction and viscosity. Arch. Ration. Mech. Anal..

[3] Müller I. (1967). On the entropy inequality. Arch. Ration. Mech. Anal..

[4] Giorgi C., Morro A. (2021). A thermodynamic approach to rate-type models of elastic-plastic materials. J. Elast..

[5] Giorgi C., Morro A. (2023). Thermodynamically-consistent modeling of ferromagnetic hysteresis. Materials.

[6] Giorgi C., Morro A. (2020). A thermodynamic approach to hysteretic models in ferroelectrics. Math. Comp. Simul..

[7] Giorgi C., Morro A. (2024). On the second law of thermodynamics in continuum physics. Thermo.

[8] Giorgi C., Morro A. (2025). Thermodynamics and nonlocality in continuum physics. Thermo.

[9] Giorgi C., Morro A. (2026). Second law of thermodynamics and strain gradient theories of elasticity. Entropy.

[10] Trovalusci P. (2026). From molecular theories to non-local continuum descriptions: Revisiting 19th century ideas for current multiscale modelling. Mech. Res. Comm..

[11] Giorgi C., Morro A., Fuhrmann J., Hömberg D., Müller W.H., Weiss W. (2025). A New Approach to Thermodynamically-Consistent Models for SMAs Advances in Continuum Physics.

[12] Giorgi C., Morro A. (2027). A thermodynamic model of shape memory alloys involving dynamic functions and constitutive entropy production. Appl. Math. Model..

[13] Talonen J., Aspegren P., Hänninen H. (2004). Comparison of different methods for measuring strain induced *α*’-martensite content in austenitic steels. Mater. Sci. Technol..

[14] Poorasadion S., Arghavani J., Naghdabadi R., Sohrabpour S. (2014). An improvement on the Brinson model for shape memory alloys with application to two-dimensional beam element. J. Intell. Mater. Syst. Struct..

[15] Lagoudas D.C. (2008). Shape Memory Alloys.

[16] Morro A., Giorgi C. (2023). Mathematical Modelling of Continuum Physics.

[17] Cisse C., Zaki W., Ben Zineb T. (2016). A review of constructive models and modeling techniques for shape memory alloys. Int. J. Plast..

[18] McBride A.T., Reddy B.D., Steinmann P. (2018). Dissipation-consistent modelling and classification of extended plasticity formulations. J. Mech. Phys. Solids.

[19] Zaki W., Moumni Z. (2007). A 3D model of the cyclic thermomechanical behavior of shape memory alloys. J. Mech. Phys. Solids.

[20] Ortin J., Delaey L. (2002). Hysteresis in shape-memory alloys. Int. J. Non-Linear Mech..

[21] Mandolino M.A., Scholtes D., Ferrante F., Rizzello G. (2023). A physics-based hybrid dynamical model of hysteresis in polycrystalline shape memory alloy wire transducers. IEEE/ASME Trans. Mechatron..

[22] Müller I. (1989). On the size of the hysteresis in pseudoelasticity. Cont. Mech. Thermodyn..

[23] Langer J.S., Grinstein G., Mazemko G. (1986). Models of pattern formation in first-order phase transitions. Directions in Condensed Matter Physics.

[24] Fried E., Gurtin M.E. (1996). A phase field theory for solidification based on a general anisotropic sharp-interface theory with interfacial energy and entropy. Physica D Nonlinear Phenom..

[25] Bollada P.C., Jimack P.K., Mullis A.M. (2012). A new approach to multi-phase formulation for the solidification of alloys. Phys. D.

[26] Boettinger W.J., Warren J.A., Beckermann C., Karma A. (2002). Phase-field simulation of solidification. Ann. Rev. Mater. Res..

[27] Singer-Loginova I., Singer H.M. (2008). The phase-field technique for modeling multiphase materials. Rep. Prog. Phys..

[28] Courant R., Hilbert D. (1953). Methods of Mathematical Physics.

[29] Penrose O., Fife P.C. (1990). Thermodynamically consistent models of phase-field type for the kinetics of phase transitions. Phys. D..

[30] Gurtin M.E. (1996). Generalized Ginzburg-Landau and Cahn-Hilliard equations based on a microforce balance. Phys. D..

[31] Landau L.D., Ginzburg V.I., Ter Haar D. (1965). On the theory of superconductivity. Collected Papers of L.D. Landau.

[32] Fabrizio M., Giorgi C., Morro A. (2006). A thermodynamic approach to non-isothermal phase-field evolution in continuum physics. Phys. D.

[33] Giorgi C. (2009). Continuum thermodynamics and phase-field models. Milan. J. Math..

[34] Giorgi C. (2015). Phase-field models for transition phenomena in materials with hysteresis. Discret. Contin. Dyn. Syst. Ser. S.

